# Endothelial ETS1 inhibition exacerbate blood–brain barrier dysfunction in multiple sclerosis through inducing endothelial-to-mesenchymal transition

**DOI:** 10.1038/s41419-022-04888-5

**Published:** 2022-05-14

**Authors:** Yan Luo, Hang Yang, Yan Wan, Sibo Yang, Jiehong Wu, Shengcai Chen, Yanan Li, Huijuan Jin, Quanwei He, Dong-Ya Zhu, Yifan Zhou, Bo Hu

**Affiliations:** 1 Department of Neurology, Union Hospital, Tongji Medical College, Huazhong University of Science and Technology, 430022 Wuhan, China; 2 Department of Clinical Pharmacology, Pharmacy College, Nanjing Medical University, Nanjing, China

**Keywords:** Multiple sclerosis, Acute inflammation

## Abstract

Blood–brain barrier (BBB) dysfunction has been recognized as an early pathological feature and contributing factor in multiple sclerosis. Endothelial-to-mesenchymal transition is a process associated with endothelial dysfunction leading to the disruption of vessel stability and barrier function, yet its functional consequence in multiple sclerosis remains unclear. Here, we demonstrated that endothelial-to-mesenchymal transition accompanied the blood–brain barrier dysfunction in several neurological disorders, especially in multiple sclerosis. The activity of transcription factor ETS1, which is highly expressed in endothelial cells (ECs) and responded to an inflammatory condition, is suppressed in the central nervous system (CNS) ECs in MS and its animal model experimental autoimmune encephalomyelitis. We identify ETS1 as a central regulator of endothelial-to-mesenchymal transition (EndMT) associated with the compromise of barrier integrity. These phenotypical and functional alterations can further induce high permeability, immune infiltration, and organ fibrosis in multiple sclerosis, thus promoting disease progression. Together, these results demonstrate a functional role of EndMT in blood–brain barrier dysfunction and propose ETS1 as a potential transcriptional switch of EndMT to target the development of multiple sclerosis.

## Introduction

Multiple sclerosis (MS) is an autoimmune disorder of the central nervous system (CNS) characterized by inflammation, demyelination, axonal degeneration, and neuron loss. Currently, immunomodulatory treatments for MS target lymphocytes to reduce recurrence, while therapeutic approaches for halting disease progression are still lacking. Myelin-specific T-lymphocytes penetrate the brain parenchyma through a damaged blood–brain barrier (BBB) to secrete cytokines that recruit peripheral leukocytes that mediate myelin destruction and neuronal damage [1, 2]. BBB disruption is an early feature in the pathogenesis of both MS and its animal model experimental autoimmune encephalomyelitis (EAE) [3, 4]. It precedes the entry of pathogenic T cells and monocytes into the CNS and has been recognized as a contributing factor to the development of disease [5, 6]. As suggested in a series of imaging studies, the global breakdown of BBB can predict the conversion from optic neuritis to MS [7]. Focal abnormality, on the other hand, correlates with active inflammation and myelin destruction in relapsing-remitting and secondary progressive MS [8]. Great efforts have been made to understand how blood–brain barrier dysfunction is regulated under pathological conditions.

The endothelial cell is a core component of the BBB in the brain and spinal cord of mammals including humans [9]. They exhibit unique properties that enable them to be a biological shelter of the CNS, including highly organized intercellular tight junctions, the absence of fenestrations and extremely low rates of transcytosis, low expression of leukocyte adhesion molecules, and a highly developed transport system [10]. A loss of the above properties causes brain edema and immune infiltration, which can further lead to clinical exacerbations.

Endothelial-to-mesenchymal transition (EndMT) has emerged as a contributing factor in the pathogenesis of vasculopathy in various diseases, including cavernous malformations [11], diabetic nephropathy [12], pulmonary arterial hypertension [13], atherosclerosis [14, 15], and age-related macular degeneration [16]. It is a process where endothelial cells (ECs) lose their typical characteristics and specialized functions, gaining a mesenchymal phenotype. On a molecular level, ECs undergoing EndMT lose endothelial markers and intercellular junctions expression, and express fibroblast and mesenchymal-specific markers [17]. This process is often accompanied by morphological and functional alterations of ECs. In the pathogenesis of various diseases, stimulations (e.g., inflammatory factors, hyperglycemia) may trigger EndMT, which in turn cause vascular dysfunction and the aggravation of disease [18–20].

In our study, we explored the GEO datasets to reveal the relevance of EndMT in BBB dysfunction and then confirmed the process in the EAE model of MS. Taking advantage of single-cell transcriptome data, we identified the molecular underpinnings of EndMT in MS. Transcription factor ETS1 was predicted to be negatively correlated with EndMT during MS. Our experiments verified that under inflammatory conditions, downregulation of ETS1 in brain ECs can induce EndMT. This is accompanied by a series of BBB events including increased permeability, immune infiltration, and perivascular fibrosis. Therefore, in the present study we investigated the possible contribution of EndMT to the pathogenesis of BBB dysfunction in MS.

## Results

### Blood–brain barrier disruption accompanied by EndMT

To identify the potential role of endothelial-mesenchymal transition in BBB dysfunction, previously published mRNA expression data derived from dysfunctional mouse CNS endothelial cells were extracted from the GEO database. The dataset was constructed with CNS ECs collected from control animals and (1) Kainic acid (KA) model of seizure, (2) experimental autoimmune encephalomyelitis (EAE) model of MS, (3) middle cerebral artery occlusion (MCAO) model of stroke, and (4) a focal cortical impact model of pediatric TBI at three time points. BBB leakage can be observed in all of the four models despite different triggers [21].

**Fig. 1:**
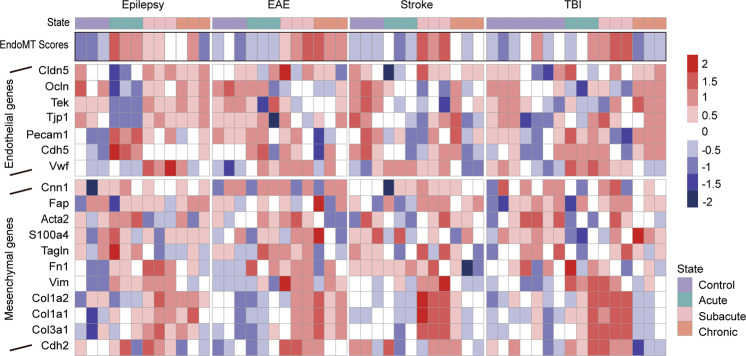
Blood–brain barrier disruption accompanied by EndMT. Heatmap depicting the BBB expression levels of endothelial and mesenchymal genes and EndMT scores in health and diseases. Three time points of four disease models including seizure, EAE, MCAO, and pediatric TBI were included. Heatmap color coding shows z-score of the log-transformed expression.

In Fig. 1, EndMT-associated gene expression and EndMT score through time points are shown for each disease. The heatmap demonstrated clear alterations in the expression profiles of endothelial and mesenchymal marker genes. Compared with controls, the expression of endothelial-specific genes decreased in all four diseases during at least one stage, including endothelial marker Cadherin 5 (*Cdh5*), Occludin (*Ocln*), and other genes encoding for junctional proteins, such as Tight junction protein ZO-1 *(Tjp1)* which are consistent with previous findings [22–24]. The upregulation of mesenchymal markers (e.g., Cadherin 2 (*Cdh2*), S100 calcium-binding protein A4 (*S100A4*), Vimentin (*Vim*)) could also be observed in the course of diseases. Other EndMT-associated transcripts including *Col1a1*, *Col3a1* encoding type I and type III fibril collagen started to increase from the subacute stage after pathology induction.

EndMT score was defined as the relative expression levels of mesenchymal and epithelial genes. A higher EndMT score reflects a more mesenchymal-like phenotype. Accordingly, a lower score indicates greater epithelial characteristics. In epilepsy, the EndMT score rose transiently and peaked at the acute phase, and remain increased for over 48 h. In EAE, stroke, and TBI, the EndMT score peaked at later time points in the subacute stage, followed by a decline during chronic stage in stroke and TBI, but continued to increase in EAE. These results indicate that endothelial-mesenchymal transition has occurred during all of the four diseases, accompanied by the occurrence of BBB dysfunction, but the severity and duration seemed to be disease-dependent.

### CNS microvascular endothelial cells undergo a mesenchymal transition in the EAE model

**Fig. 2:**
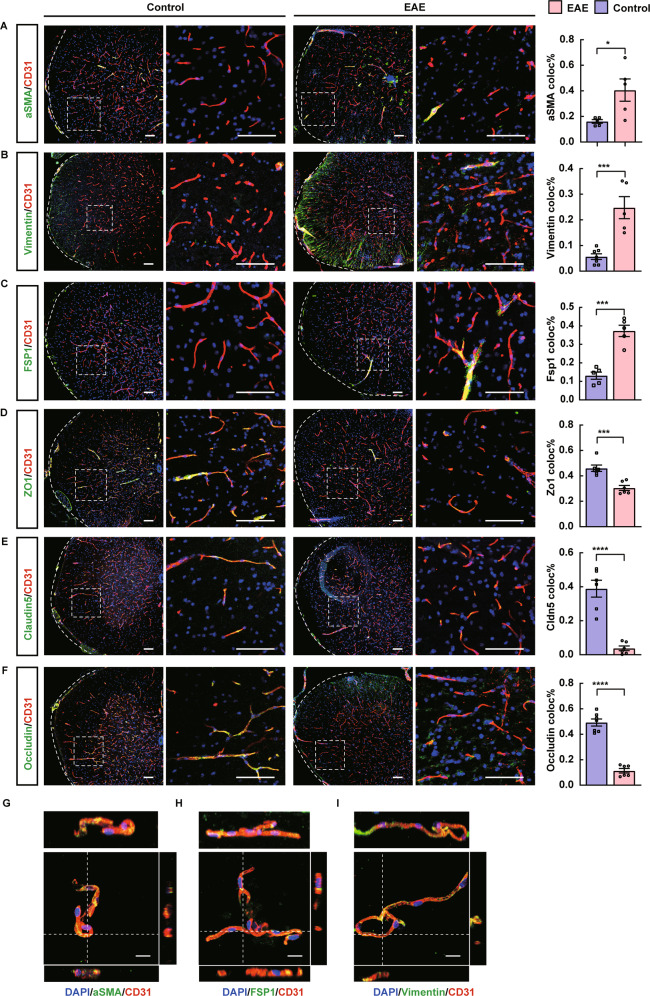
CNS microvascular endothelial cells undergo a mesenchymal transition in the EAE model. **A**–**F** Representative immunofluorescent images and quantifications of mesenchymal and endothelial marker expressions in the spinal cords of control and EAE mice (*n* > 5 per group). Scale bars: 100 μm. **G**–**I** Reconstructed Z-stack showing colocalization of mesenchymal markers and endothelial cell marker CD31 in the spinal cords of EAE mice. Scale bars: 20 μm. Mean ± SEM, 2-tailed Student’s *t* test (**A**–**F**). *P < 0.05; **P < 0.01; ***P < 0.001.

To further confirm our findings in the bioinformatic analysis, we performed immunofluorescent staining on spinal cord samples from control and EAE mouse models, and the representative images were shown in Fig. 2A–I. Mog33–35 treatment induced the gain of Fsp1 (encoded by *S100a4*) (Fig. 2C), a known sign of EndMT, and also other mesenchymal markers including αSMA (encoded by *Acta2*) and Vimentin expressions (Fig. 2A, B) by ECs [17]. We also observed the loss of endothelial markers Occludin (Fig. 2F), as well as other proteins associated with the endothelial phenotype including ZO-1 (encoded by *Tjp1*) and Claudin5 (Fig. 2D, E). Reconstructed Z-stack images showed colocalization of mesenchymal markers and endothelial cell marker CD31 in the spinal cords of EAE mice (Fig. 2G–I). These observations of increases in mesenchymal markers and reduced endothelial protein expression are consistent with previous findings [25].

### Inflammatory factors induce mesenchymal transition in CNS microvascular endothelial cells

**Fig. 3:**
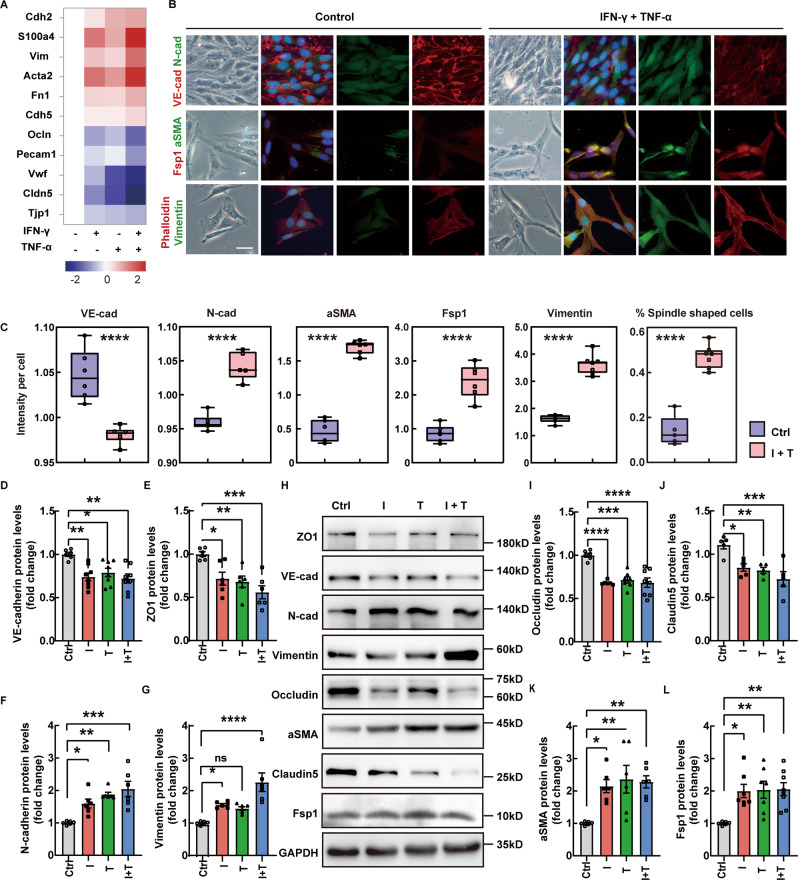
Inflammatory factors induce the mesenchymal transition of CNS microvascular endothelial cells. **A** Heatmap demonstrating mRNA expression levels of EndMT-associated genes. Values were determined by qRT-PCR and plotted as fold change relative to control. **B**, **C** VE-cadherin, N-cadherin and mesenchymal markers expression detected by immunofluorescent staining. Phalloidin staining shows the cell morphology. (Scale bar 20 µm, *n* = 5–6 independent experiments). **D**–**L** Protein levels of endothelial and mesenchymal markers were assessed by western blot and illustrated as fold change compared to controls. Ctrl, Control; I, IFN-γ; T, TNF-α; I + T, IFN-γ and TNF-α. Values were normalized to GAPDH. (*n* = 5–6 independent experiments.) Data are presented as mean ± SEM. Statistical analysis was performed using Student’s *t* test, where *p < 0.05; **p < 0.01; ***p < 0.001.

During MS, ECs undergo chronic inflammation stimulations [26]. To determine whether the mentioned stimulations can trigger the endothelial-mesenchymal transition, we stimulated bEnd.3 cells with the pro-inflammatory cytokines IFN-γ and TNF-α [27]. After 18 h of incubation, both IFN-γ and TNF-α induced a decrease in both transcription and expression of endothelial markers VE-cadherin (encoded by Cdh5) and Occludin, as well as a repress in junctional proteins ZO-1 and Claudin5. Accordingly, these two cytokines upregulated the expression of the mesenchymal genes N-cadherin (encoded by *Cdh2*), Vimentin, Fsp1. Notably, the combination of inflammatory factors showed a more significant change over mono treatment (Fig. 3A, D–L).

Downregulation of VE-cadherin and increase in N-cadherin was confirmed by immunofluorescent staining. (Fig. 3B, C) These alterations were accompanied by loss of the typical cobblestone‐like phenotype of ECs and gain of fibroblastic pattern, as well as an increase in a group of mesenchymal markers (Fsp1, aSMA, Vimentin), as revealed by microscopy (Fig. 3B, C). These results indicate chronic inflammation stimulation is a significant contributor to cerebral vascular endothelial cell EndMT during MS.

### Screening of EndMT regulatory molecules in MS

**Fig. 4:**
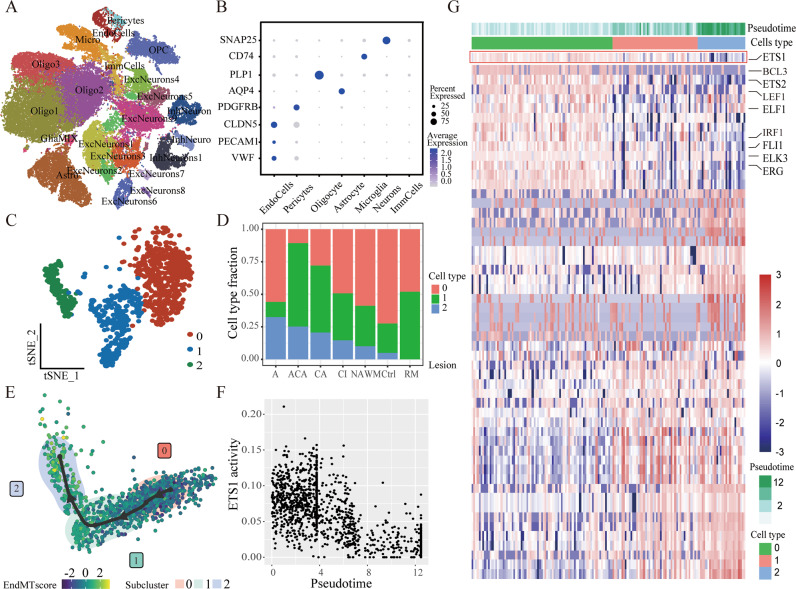
Screening of EndMT regulatory molecules in MS. **A** Clustering of single-cell RNA-seq data. Cell types were annotated according to expression of known marker genes. **B** Marker expression of each cell type. **C** Subclustering of endothelial cells. **D** Bar plot showing the relative distribution of each subcluster in different lesions. A active, ACA acute-chronic active, CA chronic active, NAWM normally appearing white matter, CI chronic inactive, Ctrl control, RM remyelinated. **E** A single-cell trajectory constructed on the endothelial cluster. ECs were ordered in pseudotime along the differentiation trajectory. **F** Scatter plot demonstrating the negative correlation of ETS1 activity score and pseudotime. **G** Heatmap demonstrating regulon activities across different cell types, with top regulators associated with EndMT process marked.

Recent research has established the belief that EndMT is a dynamic process rather than a binary process. To characterize the transitioning process and associated transcriptomic changes, previous published brain tissue single-cell transcriptome data from 21 MS patients and 21 control donors were combined to generate an integrated dataset following batch correction. As visualized in t-distributed stochastic neighbor embedding (t-SNE) plots, unsupervised clustering divided a total of assigned 62,226 cells into 22 distinct clusters across all samples (Fig. 4A). These clusters were manually identified based on the expression of known cell-type-specific markers (Fig. 4B).

To probe the heterogeneity of the ECs, we performed an unsupervised subclustering of the EC cluster using Seurat. The result showed that ECs could be further divided into three subclusters (Fig. 4C). The bar plot demonstrated the frequency of each endothelial subcluster in different conditions. Subcluster 0 was a dominant EC population in controls, detected is in 33 out of 42 samples from all two cohorts, and was characterized by endothelial markers PECAM1, VWF, and TEK. It occupies a major portion in healthy samples, but to a lesser extent in the MS samples across all time points. Of these, subcluster 2 was mostly present in MS samples, especially highly represented in the active lesions, but merely detectable in the control brain samples. This subcluster highly expressed EndMT genes VIM, TAGLN, and FN, indicating the possibility of endothelial-mesenchymal transition in pathological tissues (Fig. 4D). Moreover, we were surprised to find that subcluster 2, which represents ECs with EndMT-associated alterations, accounts for above 30% in active lesions. This result suggested that in the active lesions of MS, a considerable part of the ECs may be undergoing EndMT at the transcriptional level. However, it is well accepted that post-transcriptional and post-translational regulations also play an important role in the mesenchymal transition process. Therefore, based on our results here, it could be presumed that ECs in the MS lesion show an increased tendency towards EndMT, especially when the lesion is in an active state. But whether there are such a large number of ECs in a transitional form remains to be on a protein level.

Next, we performed the Slingshot pseudotime algorithm with the Dyno toolkit to reconstruct the putative differentiation process, which calculates the ordering of individual cells based on single-cell expression profiles. Based on this analysis, a diversion trajectory from subcluster 0 to 2 was demonstrated (Fig. 4E). The trajectory indicates the timeline of the EndMT transition, we extracted the pseudotime as an indication of the timeline of the EndMT transition, i.e., the EndMT pseudotime.

Then, we performed a single-cell regulatory network inference to calculate transcription factors (TFs) activity based on the expression of their putative target genes cell-to-cell transitioning could be explained by the kinetics of 442 regulon activities and a list of potential master regulators were identified per subtype in Fig. 4G. The activity of these regulons was plotted in the heatmap along the pseudotime trajectory.

To elucidate the molecular mechanisms driving the differentiation from a normal state towards the mesenchymal state (subcluster 0 to 2), the correlation of SCENIC activity score and pseudotime was calculated and demonstrated in Fig. 4G. The analysis revealed that a series of TFs were significantly negatively correlated with EndMT pseudotime, including ETS1, ERG, FLI1, etc. These TFs were enriched in high EC feature cells but underwent a decline of activity along the EndMT process (Fig. 4F).

### Inflammatory factors induce a decrease in the expression level of ETS1, which leads to endothelial-mesenchymal transition

**Fig. 5:**
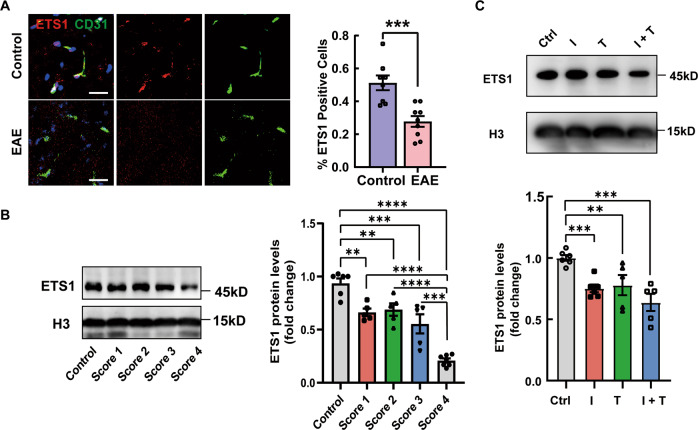
Inflammatory factors induce a decrease in the expression level of ETS1. **A** Immunofluorescence for ETS1 and CD31 (blood vessels) in the white matter of spinal cords from the control and EAE mice and quantifications. **B**, **C** Western blot detecting the nuclear ETS1 level of mouse spinal cords (**B**) and bEnd.3 cells (**C**). *n* > 5. Statistical analysis was performed using 2-tailed Student’s *t* test or one-way ANOVA, where *p < 0.05; **p < 0.01; ***p < 0.001 with post hoc Bonferroni correction.

We next aimed to validate our findings in vivo using an EAE model of MS. We quantified the percentage of ETS1+ blood vessel nuclei in lumbar spinal cord sections from control and EAE mouse models and found that ETS1 positive cell fraction decreased in CNS blood vessels during EAE (Fig. 5A) As detected by western blot, the nuclear level of ETS1 in the spinal cords significantly decreased in EAE models compared with the control mice (Fig. 5B). Animals with higher EAE scores tended to have a lower nuclear level of ETS1. In the animals with a clinical score of 4, ETS1 levels reached the lowest. Consistent with the in vivo findings, the potent inducers of EndMT, inflammatory factors TNF‐α and IFN-γ triggered a clear downregulation of ETS1 nuclear level in bEnd.3 cell line (Fig. 5C).

**Fig. 6:**
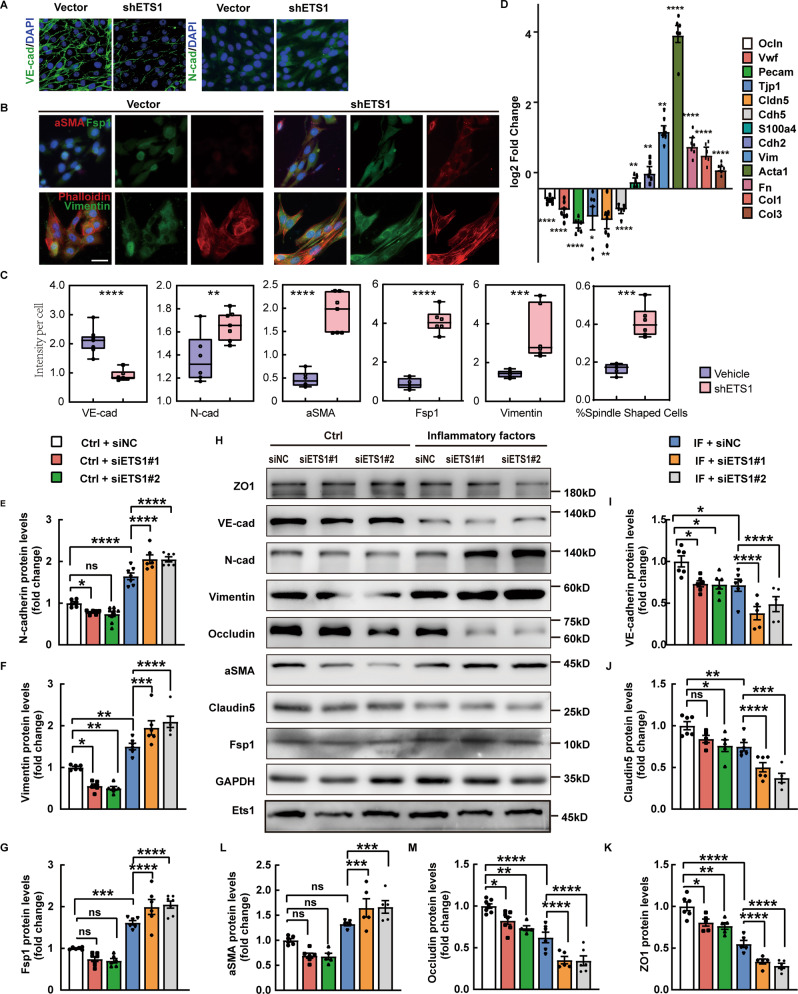
Decrease level of ETS1 leads to endothelial-mesenchymal transition under inflammation. **A** VE-cadherin and N-cadherin expression was detected by immunofluorescence (scale bar 20 µm, *n* = 5–6 independent experiments). **B**, **C** Fsp1, aSMA, Vimentin, and Phalloidin staining and quantifications in siRNA-treated versus control under inflammatory stimulations. (scale bar 20 µm, *n* = 5–6 independent experiments). **D** Fold changes in gene expression in siRNA-treated versus control under inflammatory stimulations. (*n* = 5–6 independent experiments). **E**–**M** Protein levels of endothelial and mesenchymal markers and ETS1 were assessed by western blot and illustrated as fold change compared to control. Values were normalized to GAPDH. Values were normalized to GAPDH. (*n* = 5–6 independent experiments.) IF inflammatory factors (IFN-γ and TNF-α). Data are presented as mean ± SEM. Statistical analysis was performed using 2-tailed Student’s *t* test or one-way ANOVA, where *p < 0.05; **p < 0.01; ***p < 0.001 with post hoc Bonferroni correction.

We next investigated whether ETS1 downregulation is sufficient and necessary to induce EndMT in ECs using knockdown experiments (Fig. 6D, E–M). Knockdown of ETS1 in bEnd.3 using two different siRNA constructs resulted in reduced levels of Cadherin5 and other junction proteins under inflammatory stimulations. In addition, increased expression of mesenchymal genes was observed. However, we also show that ETS1’s function is context-dependent. In the absence of inflammatory factors, siRNA-mediated ablation of ETS1 induces the elevation of Vimentin and Fsp1, which is a sign of EndMT (Fig. 6F, G). These findings support previous research into the role of ETS1 in EMT and EndMT [28, 29].

Then, we confirmed the findings using shETS1. The ablation of ETS1 resulted in the gain of a fibroblastic morphology, accompanied by increased mesenchymal markers Fsp1, aSMA, and Vimentin (Fig. 6B, C). Moreover, downregulation of VE-cadherin and an increase in N-cadherin were also observed (Fig. 6A). Altogether, our results show that downregulation of ETS1 in the context of inflammation stimulations can induce EndMT in bEnd.3 cells.

### Inhibition of ETS1 impairs blood–brain barrier integrity and increase extracellular matrix production under inflammation

**Fig. 7:**
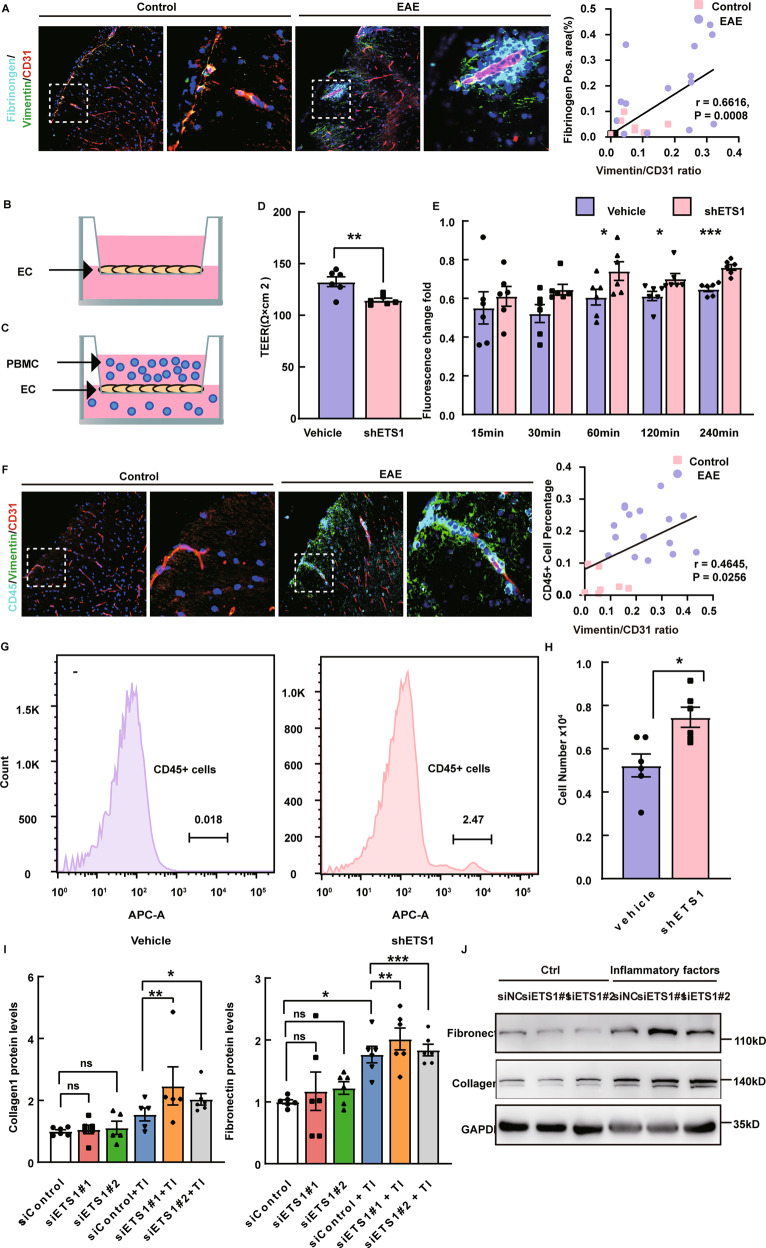
Inhibition of ETS1 impairs blood–brain barrier integrity and increase extracellular matrix production under inflammation. **A** Representative fluorescence images of Fibrinogen (cyan) and CD45+ cells surrounding microvessels undergoing EndMT in the spinal cord and analysis of correlation between EndMT fraction (represented by Vimentin/CD31) and Fibrinogen area. Scale bar, 100 μm. **B** Illustration of TEER permeability assay and FITC-dextran permeability assay. **C** Illustration of PBMC transwell assay. **D**, **E** TEER and FITC permeability of vehicle-treated (Ctrl) versus shETS1 treated endothelial cells under stimulations. (*n* = 5–6 independent experiments). **F** Representative fluorescence images of CD45+ cells (cyan) surrounding microvessels undergoing EndMT in the spinal cord, and analysis of correlation between the EndMT fraction and CD45+ cell fraction. Scale bar, 100 μm. **G** PBMC adhesion and (**H**) PBMC transmigration rate on Ctrl versus shETS1 treated endothelial cells under inflammation. (*n* = 5–6 independent experiments). **I**, **J** Protein levels of Collagen 1 and Fibronectin were assessed by western blot and illustrated as fold change compared to control. Values were normalized to GAPDH. (*n* = 5–6 independent experiments.) IF inflammatory factors (IFN-γ and TNF-α). Data are presented as mean ± SEM. Statistical analysis was performed using Student’s *t* test or one-way ANOVA, where *p < 0.05; **p < 0.01; ***p < 0.001 with post hoc Bonferroni correction.

In addition, we found a positive correlation between the level of EndMT (represented by Vimentin/CD31) and the severity of both BBB permeability and immune infiltration in spinal cords from EAE mice and controls (Fig. 7A, F). To further investigate how the loss of ETS1 affects brain endothelial barrier function, we infected bEnd.3 with lentivirus containing scramble or ETS1 shRNA to knockdown endogenous ETS1 expression. We employed a transendothelial electrical resistance﻿ (TEER) assay to measure cell monolayer permeability (greater TEER is indicative of lower permeability) (Fig. 7B) and found that ETS1-knockdown cells had significantly reduced TEER values under inflammation compared to control cells (Fig. 7D). Paracellular permeability was also abnormal in ETS1-knockdown cells, as evidenced by the increased paracellular flux of dextran particles (Fig. 7E). The above findings are consistent with the repressed expression of cell junction proteins in ETS1-knockdown cells, suggesting that ETS1 is necessary to maintain endothelial integrity during MS.

The pathogenesis of MS is closely associated with the infiltration of peripheral immune cells [30]. We adopted peripheral blood mononuclear cell (PBMC) adhesion assay and transwell assay to find out whether ETS1 inhibition could increase the infiltration of activated PBMCs isolated from EAE mice (Fig. 7C). We found that under inflammatory stimulations, PBMC adhesion exhibited an increase in ETS1-knockdown cells versus control (Fig. 7G). In addition, more PBMCs passage through the EC monolayer into the lower chamber (Fig. 7H). Our results suggested that BBB dysfunction induced by inhibition of ETS1 can lead to an increased PBMC infiltration.

By overexpression of extracellular matrix (ECM), EndMT has also been associated with fibrosis [31, 32]. In MS, excessive ECM deposition, which is typically characterized by extensive deposition of collagen and fibronectin, inhibits the regenerative capacity of oligodendrocyte progenitor cells (OPCs) and results in impaired functional recovery [33]. The cellular origin of fibrotic scar is not conclusive yet. Therefore, we examined the expression of Collagen1 and Fibronectin and observed that these ECM components were upregulated in ETS1-knockdown cells under stimulations, as evidenced by WB (Fig. 7I, J).

**Fig. 8:**
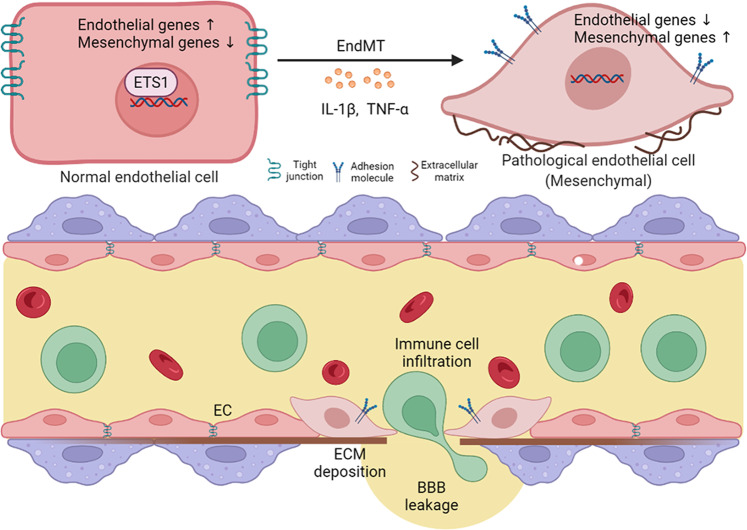
Schematic representation shows the role of ETS1 in BBB dysfunction during MS. ETS1 expression in brain endothelial cells is reduced under inflammatory stimuli, which induces the occurrence of EndMT. The process leads to increased BBB permeability, immune cell infiltration and extracellular matrix deposition.

Altogether, our observations indicate that support that inhibition of ETS1 impairs blood–brain barrier integrity and increases immune cell infiltration into the CNS system, which can lead to aggravation of MS. Moreover, EndMT microvascular ECs induced by ETS1-knockdown could be a source of fibrotic scar formation, which is thought to impair remyelination (Fig. 8).

## Discussion

EndMT is critically involved in the pathogenesis of vasculopathy in various disorders [17, 34], and regulators of EndMT are considered to have considerable potential for clinical applications in the treatment of these disorders [18]. Here we have revealed the common relevance of EndMT in BBB dysfunction of various causes including MS and found the process persisted in the EAE model of MS for all stages. There is accumulating evidence indicating that EndMT may play pivotal roles in inflammatory and fibrotic diseases, as described in atherosclerosis, vascular calcification, chronic pulmonary hypertension [14, 35–38]. A recent finding by Troletti et al. has described EndMT in the brain vessels of MS patients [25]. Their research provided important insights into vascular pathology in MS. However, despite efforts in identifying EndMT ECs during MS, little is known about EndMT and its regulators.

Starting from mRNA expression data derived from dysfunctional CNS ECs, we have revealed the common relevance of EndMT in BBB dysfunction caused by different diseases and confirmed this phenomenon in the EAE model of MS. These results are consistent with the observations of Troletti et al. Notably, we have identified a group of EndMT ECs characterized by reduced ETS1-driven gene expression in MS brain ECs. These results suggested that downregulation of the transcription factor ETS1 could potentially induce EndMT in MS. This prediction was proved in the following research using cell and animal models. In the end, we discussed the pathological results associated with reduced ETS1 and the subsequent EndMT and propose that ETS1 may be a possible target for the treatment of MS.

In the present research, we have identified ETS1 as a core regulator of EndMT in MS through reanalysis of single-cell transcriptome data of pathological tissues. ETS1 is a member of the ETS family of transcription factors [39, 40]. It plays a critical role in embryonic and early postnatal development [41]. In the adult, Ets1 is highly expressed in immune cells, and also detectable in several other cell types including ECs. Intriguingly, single-nucleotide polymorphism (SNP) rs3809006 of ETS1 showed a significant association with MS susceptibility [42]. Consistent with this clue, previous studies have demonstrated that reduced ETS1 level leads to an aggravation of EAE. They have been focused on the immune cells and identified ETS1 as a negative regulator of Th17 differentiation, which is a key participant in autoimmune diseases including MS [43–45]. Here, we reconsider whether endothelial ETS1 levels play a role in disease pathogenesis. Based on our observations, Ets1 levels decreased in brain ECs during MS. Under inflammatory conditions, downregulation of ETS1 in CNS ECs can induce EndMT and a series of BBB events including increased permeability, immune infiltration, and perivascular fibrosis. Clinically, our findings increase the translational value of ETS1 as a treatment target.

The ETS family transcription factors are believed to be master regulators of EC [29]. In contrast to the other family members like Erg, which are believed to preserve the endothelial characteristics and resist mesenchymal transition, ETS1 has merely been reported to induce EndMT and epithelial to mesenchymal transition [28, 29]. Our observations showed the context-dependent role of ETS1 in EndMT. Notably, other transcription factors like IRF1 also demonstrated a similar context-dependent role in EndMT [46]. This is probably a result of a complex interaction of transcription factors and chromosomes. The findings of the present study might help to extend previous knowledge of ETS1 and its function in brain ECs during MS.

The present study found that EndMT accompanies the occurrence of BBB dysfunction during many neurological diseases. It might indicate that different upstream lead to convergent downstream pathways under attacks. However, since the severity and duration of this process are highly cause-dependent, there is still a lot to be clarified in the future. On the other hand, this leaves room for exploring disease-specific treatments.

Overall, we have shown that EndMT is closely associated with BBB dysfunction in MS. Transcription factor ETS1 is a key regulator of this process. Under an inflammatory condition, the inhibition of ETS1 can promote EndMT in CNS ECs. This phenotypic alteration enhanced the expressions of mesenchymal markers, while repressing the expressions of proteins that are essential to EC integrity. Our data indicate that EndMT triggered by ETS1 inhibition can increase immune cell infiltration and broaden fibrotic regions in perivascular areas, which contributes to EAE pathology. Although our study showed that decreased ETS1 levels can induce EndMT in MS, it remains challenging to fully elucidate the underpinning mechanisms. Thus, we expect further research to fill substantial gaps in current knowledge.

## Methods

### Animals

All experiments with mice were approved by the medical ethics committee of Tongji Medical College, Huazhong University of Science and Technology, Wuhan, China, and were performed following the Guide for the Care and Use of Laboratory Animals (National Institutes of Health, eighth edition, 2011). Female C57BL/6 mice of age 9 to 13 weeks were purchased and fed in the specialized facilities of the Animal Care and Use Committee of Tongji Medical College, Huazhong University of Science and Technology. Animals were randomly divided (table of random numbers) into control and EAE groups.

### MOG-induced EAE model

Female C57BL/6 mice of age 9 to 13 weeks were used to induce chronic monophasic EAE. PBS solution of Myelin oligodendrocyte glycoprotein (MOG) peptide 35–55 (3 mg/mL) (Synthetic Biomolecules) was emulsified in and Complete Freund’s Adjuvant (CFA) containing 4 mg/mL heat-killed mycobacteria tuberculosis H37Ra (Invivogen, China). Each mouse was immunized with 200ul MOG/CFA or CFA only (as negative control) by flank subcutaneous injections. 500 ng of pertussis toxin (Sigma-Aldrich, USA) was administered intraperitoneally at 0 and 2 days post-immunization (dpi). Disease progression was monitored daily and clinical scores were defined as follows: [0 = no obvious symptoms, 1 = loss of tail tonicity, 2 = weakness of hind legs, 3 = hindlimb paralysis, 4 = limbs paralysis, 5 = moribund or death]. Grade 5 mice were removed from the study. Unless otherwise stated, mice were sacrificed for experiments when the disability score leveled off for 1–2 days, ~17dpi. Besides, mice that reached a clinical score of 4 were sacrificed immediately for animal welfare considerations.

### Immunofluorescence staining

Mice were anesthetized and perfused with PBS/4% paraformaldehyde (Electron Microscopy Sciences). The lumbar enlargements of the spinal cord and the brain tissues were collected and fixed with 4% paraformaldehyde for 16 h at 4 °C, dehydrated with a sucrose solution, subsequently embedded in OCT, and then cut into 20 μm sections. Sections were stained with an H&E staining kit (Beyotime Biotechnology, China) and toluidine blue LFB staining solution (Solarbio, China) and matched areas were selected for immunostaining.

After washing with PBS, tissue sections were blocked with 10% donkey serum in PBST (0.05% Triton X-100 in PBS) for 30 min at room temperature and then incubated overnight at 4 °C with primary antibodies. The antibodies used are shown in Table S2. After washing, the sections were incubated with the following secondary antibodies at 1:100 concentration for 2 h at room temperature: Alexa Fluor 594 or 647 conjugated donkey anti-rabbit IgG (H + L) (Jackson ImmunoResearch, West Grove, PA) or Alexa Fluor 488 or 647 conjugated donkey anti-mouse IgG (H + L) (Jackson ImmunoResearch; Antgene, China). Nuclei were stained with 4′,6-diamidino-2-phenylindole (DAPI). Fluorescent images were captured at ×20–×40 magnification with a Nikon A1Si confocal microscope (Nikon, Japan).

ImageJ was used for downstream quantitative analysis of immunofluorescent captures. More specifically, after the auto threshold process of 8-bit pictures, CD31-positive and the target protein-positive regions were detected respectively. Co-immunostaining regions of CD31 and the target protein were determined with the AND operation. The ratio of CD31+ target+ area to the CD31+ area was calculated as the quantification index. For cell staining, the average fluorescence intensity was calculated by subtracting the total nucleus number from the total fluorescence intensity of the target protein.

### RNA interference

siRNA for mouse ETS1 and a negative control were obtained from OBiO Technology (Shanghai). bEnd.3 cells were purchased from ATCC and transfected at 40% confluence with a final concentration of 25 nM either ETS1 siRNA or a scramble control using lipofectamine3000 transfection reagent (Invitrogen, USA) according to the manufacturer’s instructions. bEnd.3s were cultured for 24 h after transfection and then stimulated with the presence or absence of 500 U/ml TNF-α and IFN-γ for the indicated time. The efficiency of gene silencing of ETS1 was determined by western blot.

### RNA extraction and real-time quantitative PCR

Total RNA was extracted with the total RNA extraction reagent (Vazyme). The cDNA was synthesized using HiScript III-RT SuperMix (Vazyme) according to the manufacturer’s instructions. Real-time PCR was performed using SYBR Green (Vazyme) and Reactions were run using the CFX Real-Time PCR System (Biorad). Gene expression was analyzed using the comparative Ct method and normalized to the Gapdh level. The primer sequences used are listed in Table S1.

### Western blot analysis

Cells were lysed with the RIPA buffer containing protease inhibitors. Protein extracts were fractionated in SDS-PAGE and transferred onto a polyvinylidene difluoride (PVDF) membrane (Millipore). The membranes were blocked at room temperature in 5% BSA TBST buffer for 1 h, then incubated at 4 °C with the indicated primary antibodies overnight. After washing with TBST buffer, membranes were incubated with secondary antibodies for 2 h at room temperature. The target proteins were visualized by an enhanced chemiluminescence system and analyzed with ImageJ. The antibodies used are shown in Table S2.

### Lentivirus packaging and transfection

Lentivirus containing ETS1 short hairpin RNAs (shRNAs) were purchased from OBiO Technology (Shanghai). bEnd.3 cells were infected with control shRNA LV or ETS1 shRNA LV supernatant with HitransG A infection solution (1:24; Genechem, China), and stable cells were selected with puromycin (4 mg/ml; Sigma-Aldrich) and confirmed by immunoblotting.

### Transendothelial electrical resistance measurement

Transendothelial electrical resistance (TEER) was measured using a resistance meter (ERS-2, Millipore, Billerica, MA, USA) after 12 h incubation with TNF‐α and IFN-γ. We equilibrated the cultures at room temperature for 20 min before detection. The resistance can characterize the formation of a tight endothelial cell monolayer and was calculated as follow [47]: TEER (Ω × cm^2^) = [(resistance of insert with cells − resistance of blank insert) × surface area of the insert].

### Flow cytometry analyses

The second generation of primary pericytes in different groups was lysed with 0.25% trypsin without EDTA. Cells were collected and washed in ice-cold PBS twice. Cell suspensions were incubated for 30 min with anti-CD45 APC (Biolegend, USA) in FACS buffer (Becton Dickinson, USA), washed, and incubated with goat anti-rabbit IgG in FACS buffer for 30 min. The cells were then suspended in the same buffer for analysis. All data were collected and analyzed by FlowJo software (Treestar, Ashland, OR, USA).

### External data sources

We obtained the human MS and Control brain datasets from publicly available repositories. Raw data (fastq files) from Schirmer et al. [48] were accessed at the Sequence Read Archive (SRA) at the accession number PRJNA544731. UMI count matrices from Jäkel et al. [49] were retrieved from GEO at the accession number GSE118257.

### Processing of scRNAseq data

FASTQ files were processed by CellRanger v3.0 for decoding, alignment, quality filtering, and UMI counting. Empty droplets were detected and removed based on a similar expression profile of the ambient solution with the package DropletUtils [50, 51].

### Quality control for the expression matrix

The initial datasets contained 55,121 cells with a total of 33,538 genes (Schirmer) and 17,801 cells with a total of 21,581 genes (Jäkel) respectively. and pre-processed independently. We manually excluded low-expressed genes and low-quality cells in the initial quality control (QC) step according to the following quality measures: (i) genes expressed in fewer than three cells were discarded, (ii) cells with less than 150 genes and 300 nUMIs, and cells that express >3500 genes were removed from downstream analysis, (iii) cells that had >0.10 mitochondrial percentage among their expressed genes. After quality controls, 13,959 and 48,267 cells remained (Jakel and Schirmer).

### Doublet identification and removal

Potential doublet cells were identified based on a doublet score calculated with the scran package function doublet Cells. This function simulates putative doublet expression profile and computes the normalized density of simulated doublets around each cell as the doublet score. Cells with a doublet score of > = 1.5 were excluded from downstream analysis.

### Lognormalized and batch correction

For further analyses, the data was loaded into Seurat (version 3.1.5) [52, 53] We implemented the SCTransform function (regularized-negative binomial modeling) to normalize and log-transformed the expression data per sample, as well as to regress out variations arising from the library size, cell cycle and percentage of mitochondrial genes. The top 3000 highly variable genes (HVGs) were selected. Batch-effects were corrected using the integration function on the first 30 PCs computed from the identified variable genes only.

### Dimensionality reduction, clustering, and data visualization

Dimensional reduction of the integrated data was achieved by principal component analysis (PCA) using top 3000 highly variable genes and the subsequent t-SNE embedding for visualization. We ranked principal components (PC) in an “elbow plot” according to the percentage of variance explained by each of them and selected the first 20 PCs as meaningful upon visual inspection (for downstream analysis). Cells were clustered with a graph-based method in Seurat with a resolution of 0.8. Overall, we assigned 62,226 cells into 22 different cell clusters.

Subclustering was further performed using 15 PCs. Cells in the EC cluster were extracted and reclustered in the same procedure with perplexity parameter set to 0.1 [54]. A dot plot was utilized to visualize the distribution of gene expression among clusters. Feature plots were drawn to color single cells according to their gene expression on a two-dimensional tSNE space.

Then, differentially expressed markers for each cluster were calculated against all other clusters using a Wilcoxon Rank Sum test with default parameters of the FindAllMarkers function, and *p* values were adjusted based on the Bonferroni correction.

We extracted previously characterized marker genes of each brain cell type from recent publications [55–58] and visualized them by the t-SNE plot. and compare them with the differentially expressed markers. Cell types were annotated based on the expression of these marker genes and the assigned cell identity was confirmed by significant overlap within the cluster marker genes and known markers of that cell type.

### Reclustering of the cell subtypes

To identify subclusters within cell subtypes, we separately reanalyzed cells that belonged to different cell types. Specifically, we reselected the HVGs for each cell subtype as described above and then applied PC analysis to the selected HVGs for dimensionality reduction. Batch effect correction and UMAP dimensionality reduction using default and graph-based clustering cell reclustering were also performed as described above.

### Inference of regulons and their activities using SCENIC

SCENIC v1.2.4 [59] were used to infer transcription factor activity in each cell following the standard workflow. After an initial filter of the expression matrix, gene sets co-expressed with transcription factors were identified based on the calculated correlation networks between TFs and potential targets. Next, motifs were identified based on the hg38 cisTarget databases and Co-expression modules were excluded unless the transcription factor motif was enriched among its targets. Finally, regulon activity was computed using the AUCell (Area Under the Curve) package. The correlation of SCENIC activity score and pseudotime was calculated using the “cor” function with the Pearson method in R.

### Trajectory inference (TI) analysis

The trajectory inference analysis was conducted with the Dyno R package v0.1.2 [60]. Considering its accuracy and usability, Slingshot was selected and applied with default parameters on the identified EC cells to determine the potential lineage differentiation [61]. To visualize the trajectory, cells were plotted on the output dimensionality reduction in order. Pseudotime was calculated for each cell as the distance to the root of the trajectory.

### Calculation of EndMT score and heatmap generation

According to the original research, ECs were enriched at 3 h (acute), 48 h (subacute), and 1 month (chronic) post-immunization of Seizure. For permanent MCAO and pediatric TBI models, the three different time points were taken at 24 h (acute), 72 h (subacute), and 1 month (chronic) after surgery. And for EAE models, acute time points were determined as the first day that animals displayed a loss of 1 g body weight. Subacute time points were taken when the disability score leveled off for 1 d. Chronic time points were taken 14 days after peak score (Munji et al. [21]). For each sample, we obtained the expressions of a set of endothelial-associated genes, as well as a set of mesenchymal genes (listed in Fig. 1). The geometric mean of each set of genes in a particular cell was calculated as the endothelial score or mesenchymal score. EndMT score is defined as the ratio of the mesenchymal score and endothelial score. Briefly, we calculated the EndMT score for each sample using the following formula:

EMT score = Geometric mean of mesenchymal gene expression (Cnn1, Fap, S100a4, Tagln, Fn1, Vim, Col1a2, Col1a1, Col3a1, Cdh2, Acta2)- Geometric mean of endothelial gene expression (Cdh5, Cldn5, Tek, Pecam1, Tjp1, Vwf).

For heatmaps, z-score of the log-transformed expression of interest genes were plotted using the “pheatmap” package in RStudio.

### Statistical analysis

All values are presented as mean ± SEM unless otherwise stated. Student *t* test was performed for comparison between two groups. One-way analysis of variance was used for multiple comparisons. Correlation analysis was performed using the Pearson correlation test. *P* < 0.05 was considered statistically significant. All analyses were conducted using GraphPad Prism version 8.0 and RStudio version 1.2.5033. Sample size was determined based on previous studies and not based on any statistical analysis. Animals that died before the scheduled times were excluded from further analysis. No other data were excluded from the analyses.

## Supplementary information

Original Data File

Author Contribution form

co-authors replies confirming that they agree to the changes in author list

Checklist

Supplementary Tables

## Data Availability

The data that support the findings of this study are publicly available from the GEO database. No extra data was generated in this study.
