# Supplementary material for: Endothelial ETS1 inhibition exacerbate blood–brain barrier dysfunction in multiple sclerosis through inducing endothelial-to-mesenchymal transition
Source: Cell Death Dis. 2022 May 14;13(5):462. doi: 10.1038/s41419-022-04888-5 (PMC9107459; doi:10.1038/s41419-022-04888-5)
Supplement: Supplementary file 1 — Original Data File [file 41419_2022_4888_MOESM1_ESM.pptx]

## Slide 1
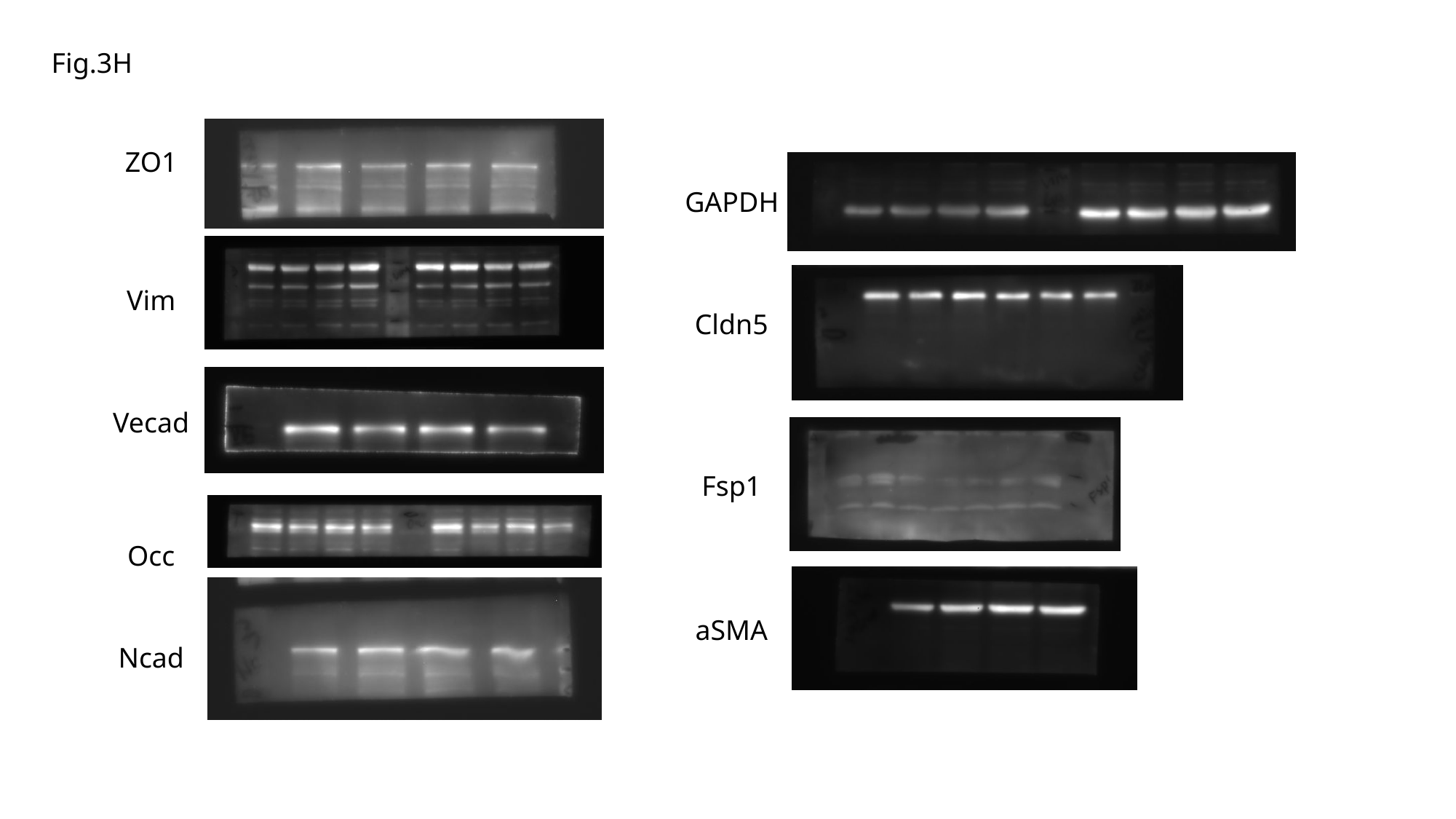

Fig.3H
ZO1
GAPDH
Vim
Cldn5
Vecad
Fsp1
Occ
aSMA
Ncad

## Slide 2
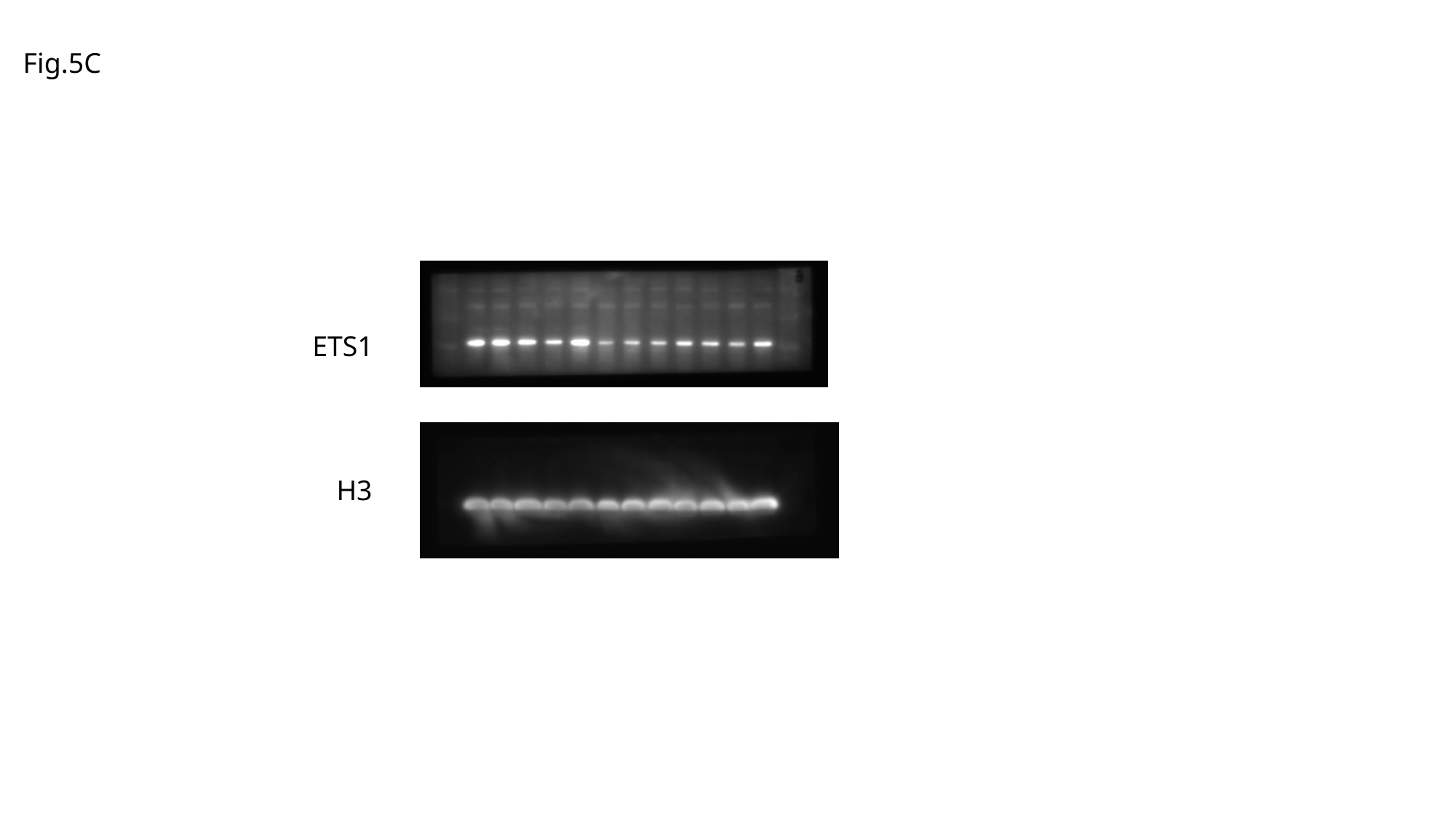

Fig.5C
ETS1
H3

## Slide 3
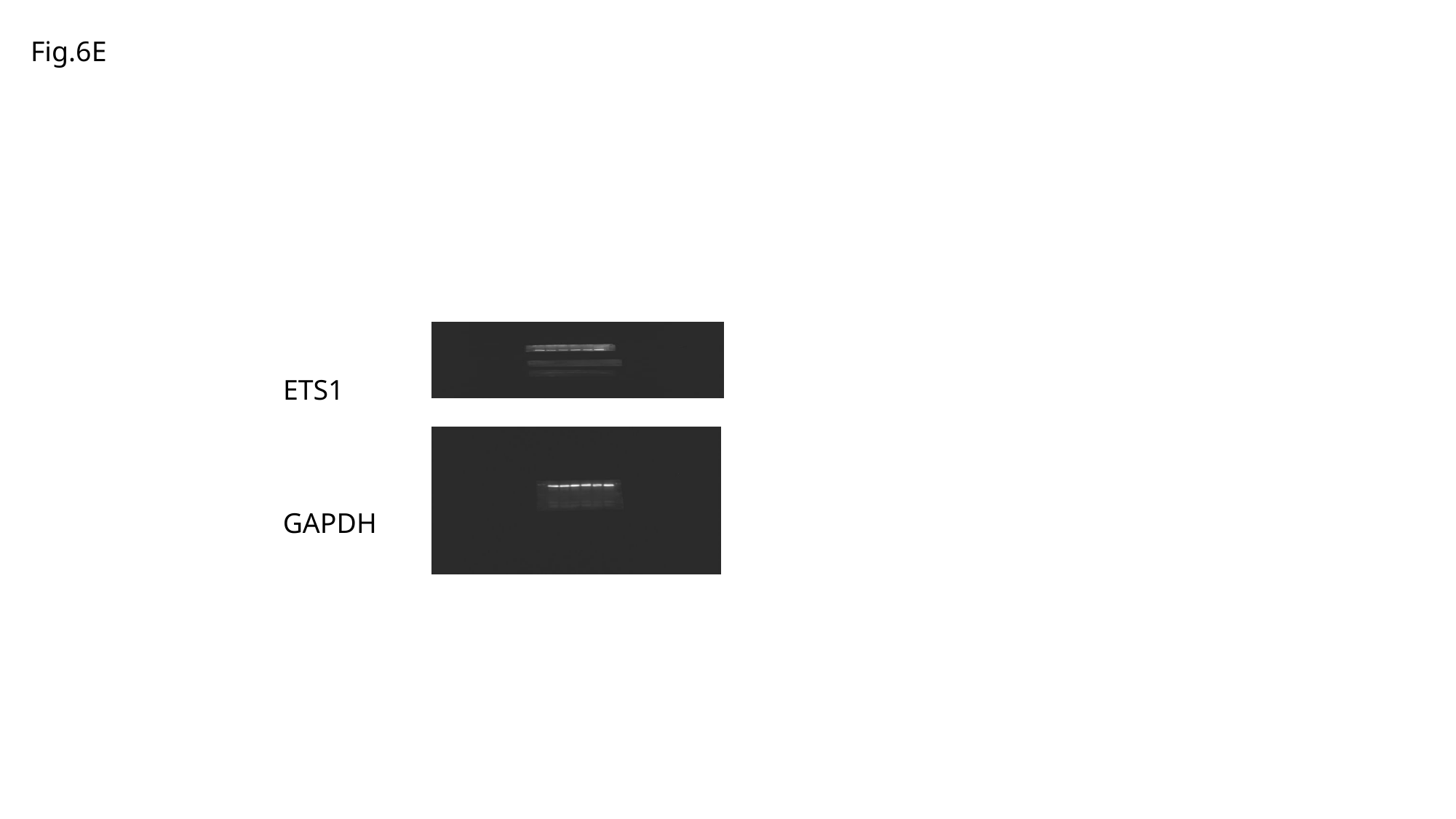

Fig.6E
ETS1
GAPDH

## Slide 4
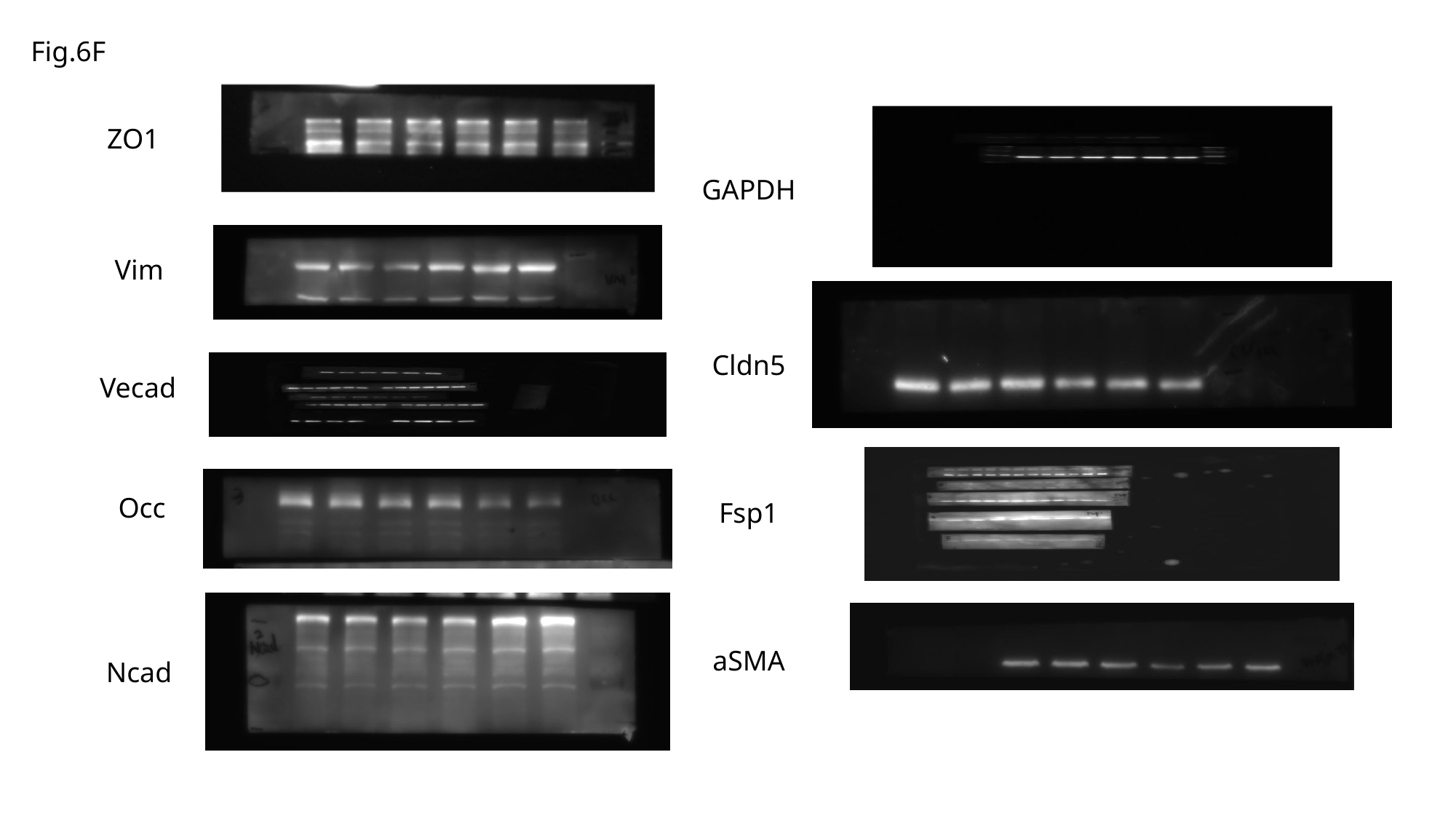

Fig.6F
ZO1
GAPDH
Vim
Cldn5
Vecad
Occ
Fsp1
aSMA
Ncad

## Slide 5
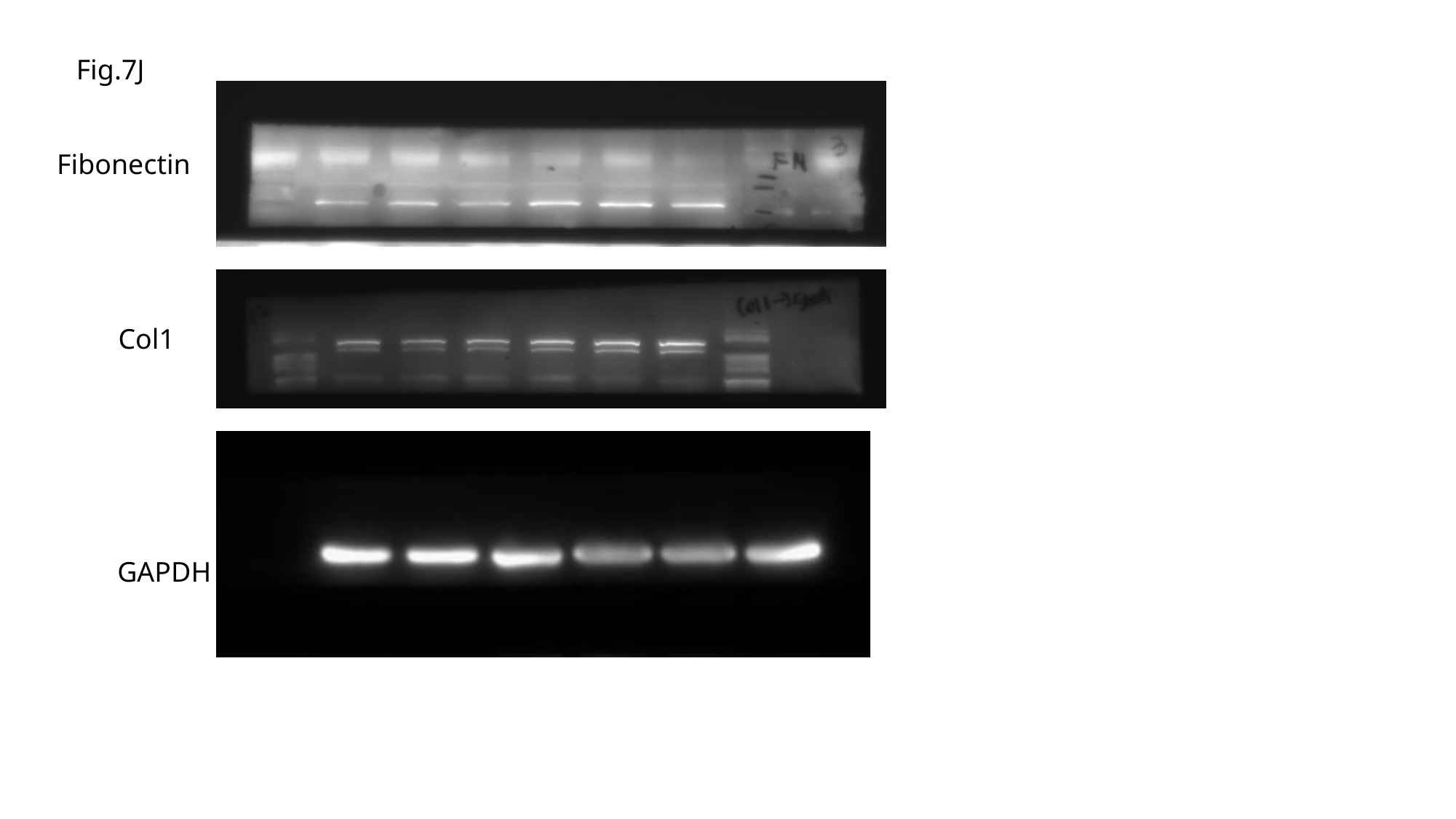

Fig.7J
Fibonectin
Col1
GAPDH
