# Supplementary material for: Endothelial ETS1 inhibition exacerbate blood–brain barrier dysfunction in multiple sclerosis through inducing endothelial-to-mesenchymal transition
Source: Cell Death Dis. 2022 May 14;13(5):462. doi: 10.1038/s41419-022-04888-5 (PMC9107459; doi:10.1038/s41419-022-04888-5)
Supplement: Supplementary file 2 — Author Contribution form [file 41419_2022_4888_MOESM2_ESM.pdf]

DECLARATION OF CONTRIBUTIONS TO ARTICLE

ADMC

Manuscript Number: CDDIS-21-4584RR

Journal Name: *Cell Death & Differentiation* (the 'Journal')

Proposed Title of the Contribution:

Endothelial ETS1 inhibition exacerbate blood brain barrier dysfunction in multiple sclerosis through inducing endothelial to mesenchymal transition (the 'Contribution)

Author(s):

Yan Luo, Hang Yang, Yan Wan, Sibo Yang, Jiehong Wu, Shengcai Chen, Yanan Li, Huijuan Jin, Quanwei He, Dong-Ya Zhu, Yifan Zhou, Bo Hu (the 'Authors')

For all *CDD* articles, each person named as an author in the published version must be able to show he or she has contributed substantially to the article.

Authorship credit should be based on 1) substantial contributions to conception and design, acquisition of data, or analysis and interpretation of data; 2) drafting the article or revising it critically for important intellectual content; and 3) final approval of the version to be published. Authors should meet conditions 1, 2 and 3.

Any person who cannot be shown to have made a substantial contribution to the article cannot be listed as an author in the final version. The name of any person who is deemed to have made a minor contribution can, however, appear in the Acknowledgments section of the article.

Please complete the table below to indicate the contributions of all named authors to the manuscript.

Author Full Name:	Specification of Contribution to the Manuscript:
Yan Luo	performed the experiments;performed interpretation of data, and statistical analysis, and writing of the paper;
Hang Yang	performed the experiments;performed interpretation of data, and statistical analysis, and writing of the paper;
Yan Wan	performed study concept and design. performed the review and revision of the paper; help to revise the article
Sibo Yang	performed the experiments;performed interpretation of data, and statistical analysis, and writing of the paper;
Jiehong Wu	provided technical and material support.
Shengcai Chen	provided technical and material support.
Yanan Li	provided technical and material support.
Huijuan Jin	provided technical and material support.
Quanwei He	provided technical and material support.
Dong-Ya Zhu	provided technical and material support.
Yifan Zhou	performed study concept and design. performed the review and revision of the paper
Bo Hu	performed study concept and design. performed the review and revision of the paper

Please complete the table below to indicate the contributions of all named authors to the figures.

Figure 1:

Yan Luo, Hang Yang, Yan Wan, Yifan Zhou, Bo Hu

Figure 2:

Yan Luo, Hang Yang, Jiehong Wu, Yifan Zhou, Bo Hu

Figure 3:

Yan Luo, Hang Yang, Sibo Yang, Huijuan Jin, Yifan Zhou, Bo Hu

Figure 4:

Yan Luo, Hang Yang, Shengcai Chen, Yifan Zhou, Bo Hu

Figure 5:

Yan Wan, Sibo Yang, Yanan Li, Quanwei He, Dong-Ya Zhu, Yifan Zhou, Bo Hu

Figure 6:

Yan Luo, Yan Wan, Sibo Yang, Yifan Zhou, Bo Hu

Signed for and on behalf of the Author(s):	Print Name:	Date:
Bo Hu	Bo Hu	22/4/13
