# Supplementary material for: Endothelial ETS1 inhibition exacerbate blood–brain barrier dysfunction in multiple sclerosis through inducing endothelial-to-mesenchymal transition
Source: Cell Death Dis. 2022 May 14;13(5):462. doi: 10.1038/s41419-022-04888-5 (PMC9107459; doi:10.1038/s41419-022-04888-5)
Supplement: Supplementary file 3 — co-authors replies confirming that they agree to the changes in author list [file 41419_2022_4888_MOESM3_ESM.docx]

| Yifan Zhou | 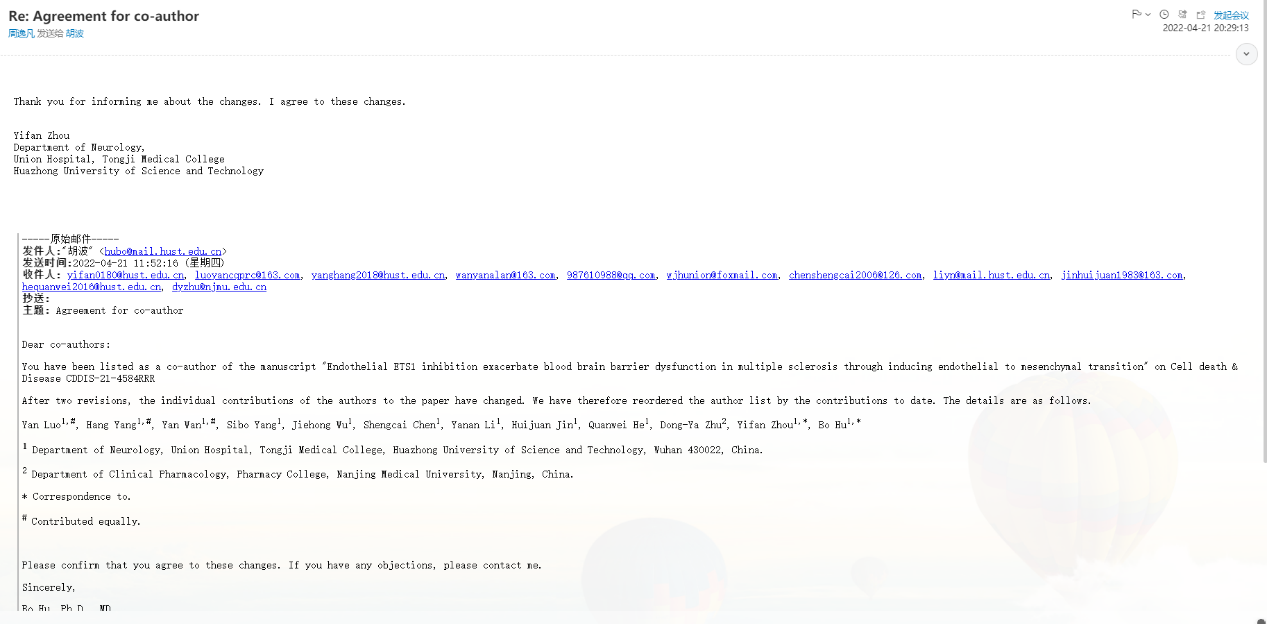 |
| --- | --- |
| Hang Yang | 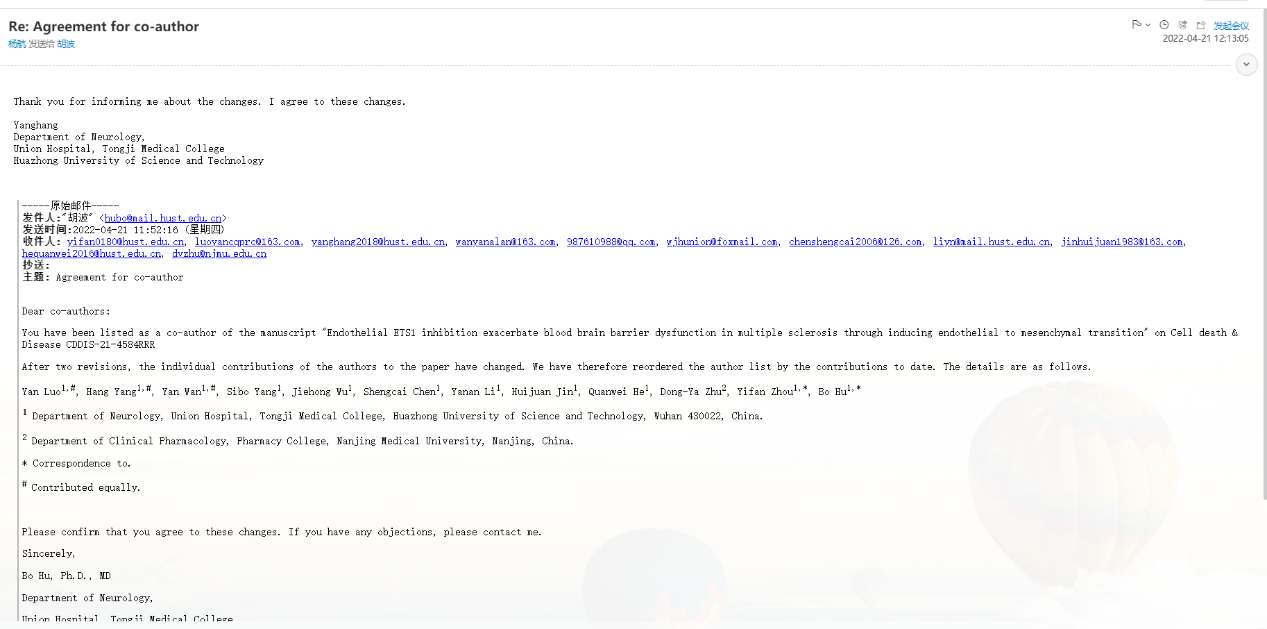 |
| Yan Luo | 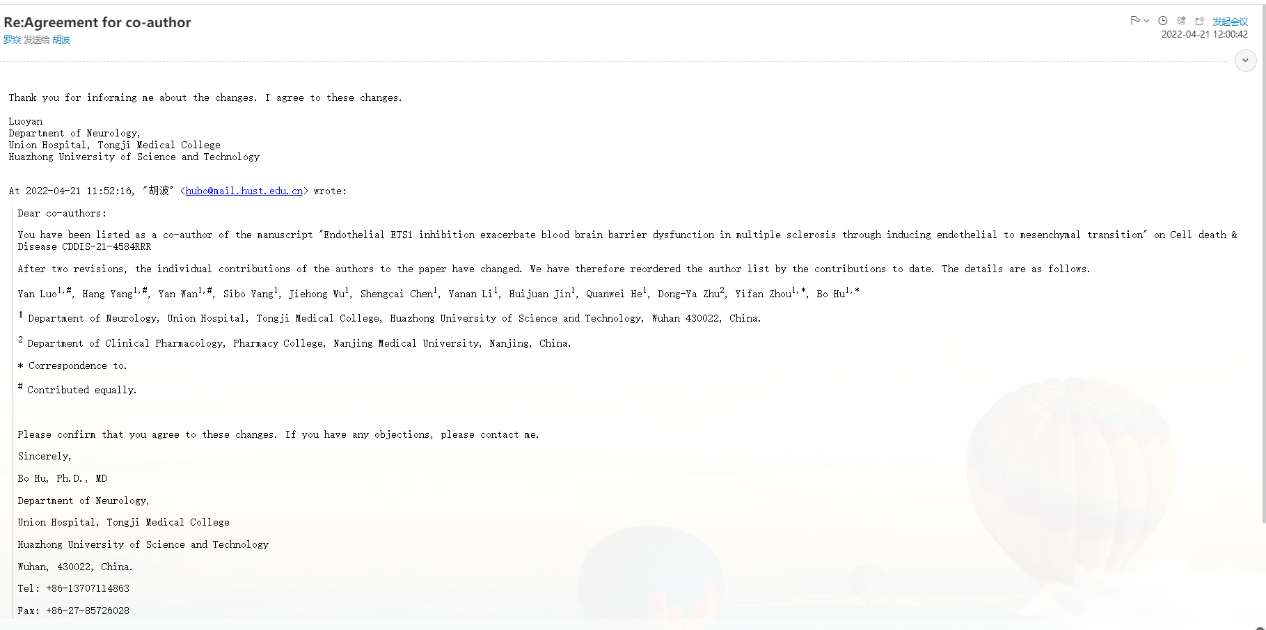 |
| Yan Wan | 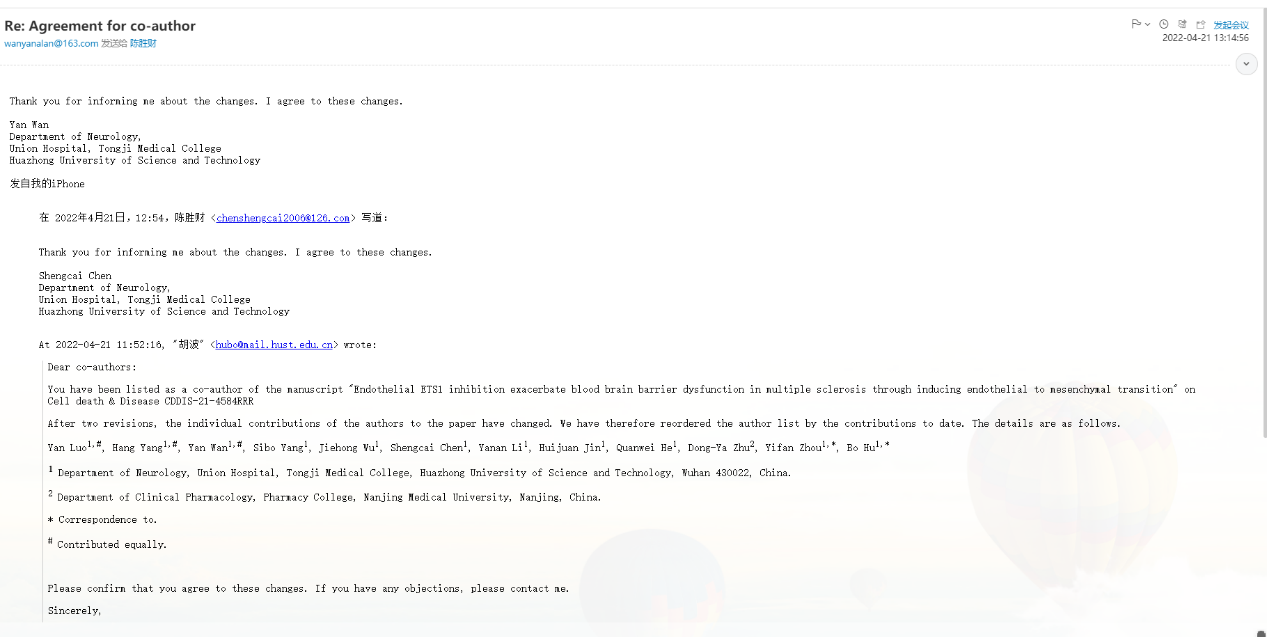 |
| Sibo Yang | 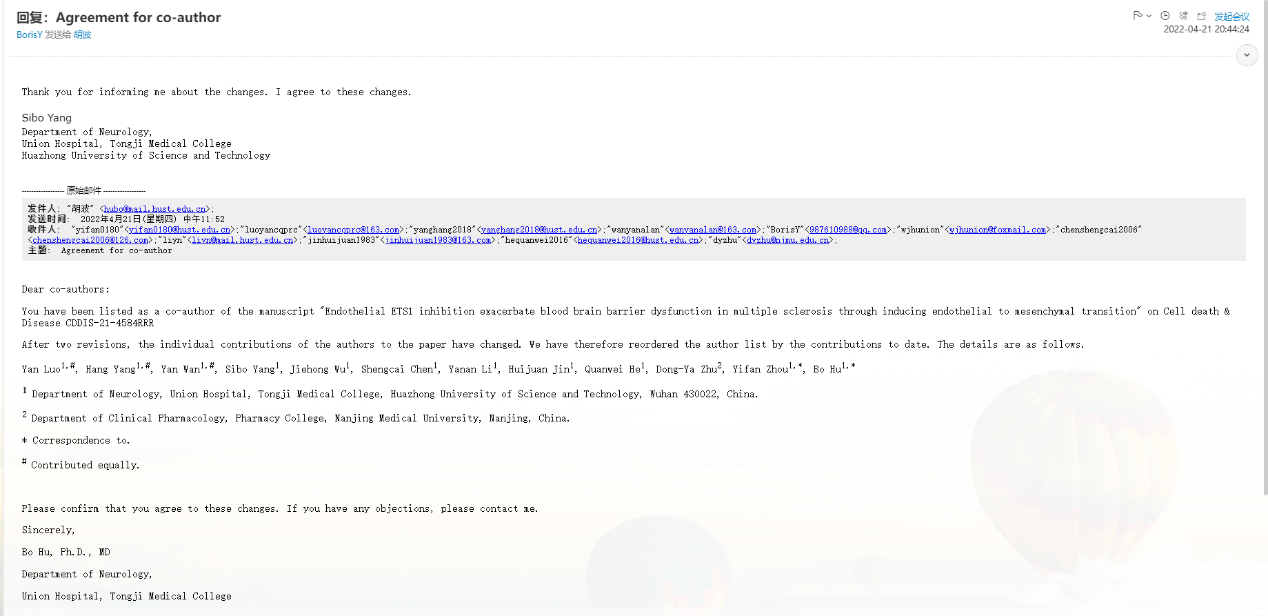 |
| Jiehong Wu | 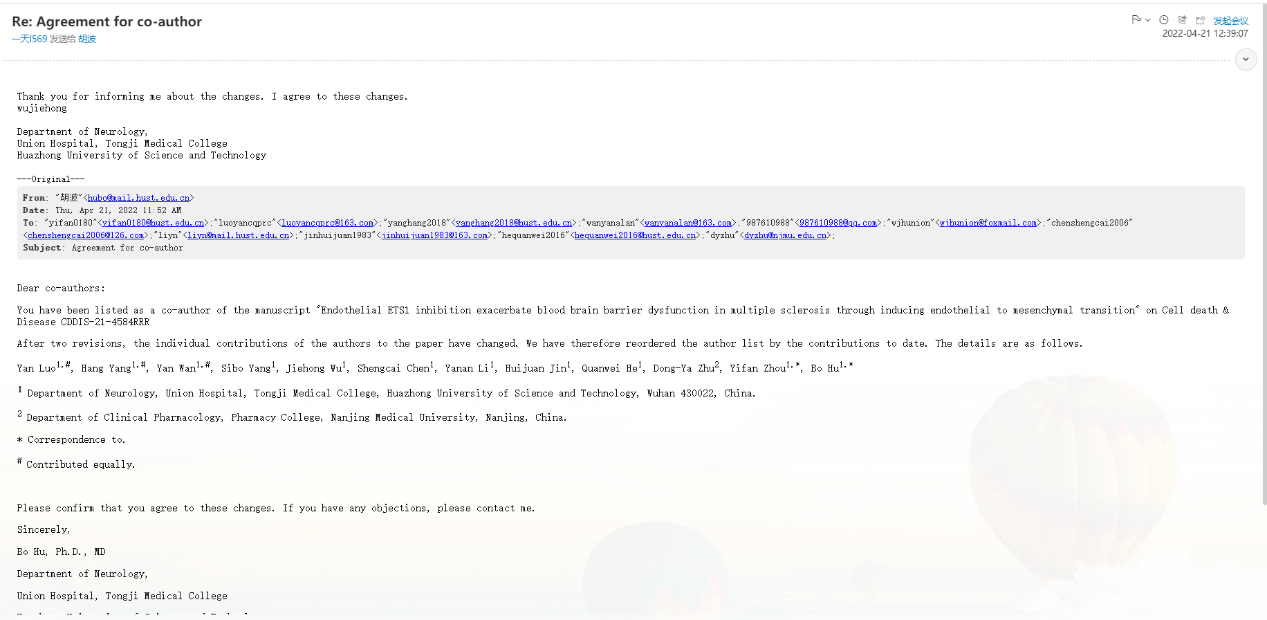 |
| Shengcai Chen | 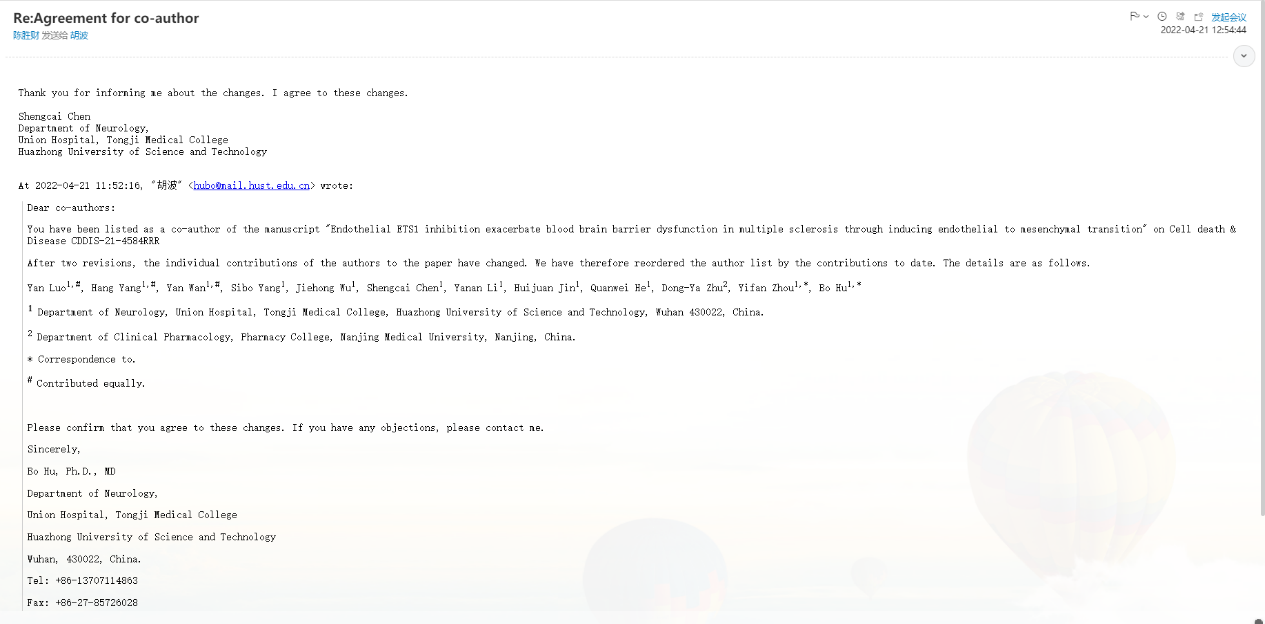 |
| Yanan Li | 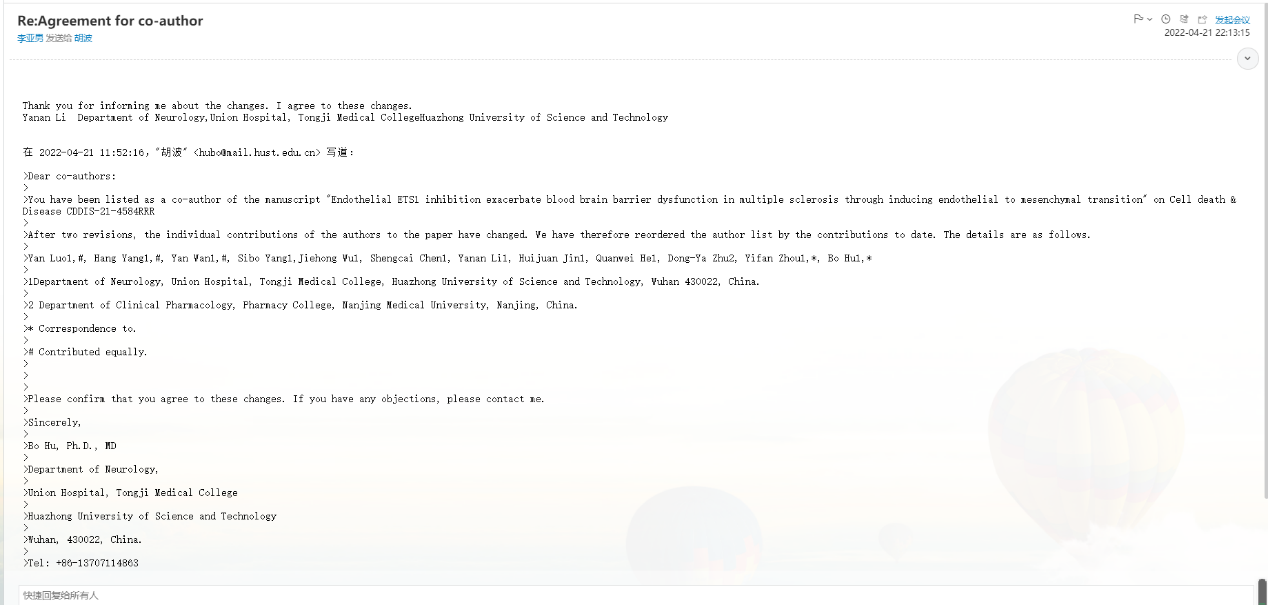 |
| Huijuan Jin | 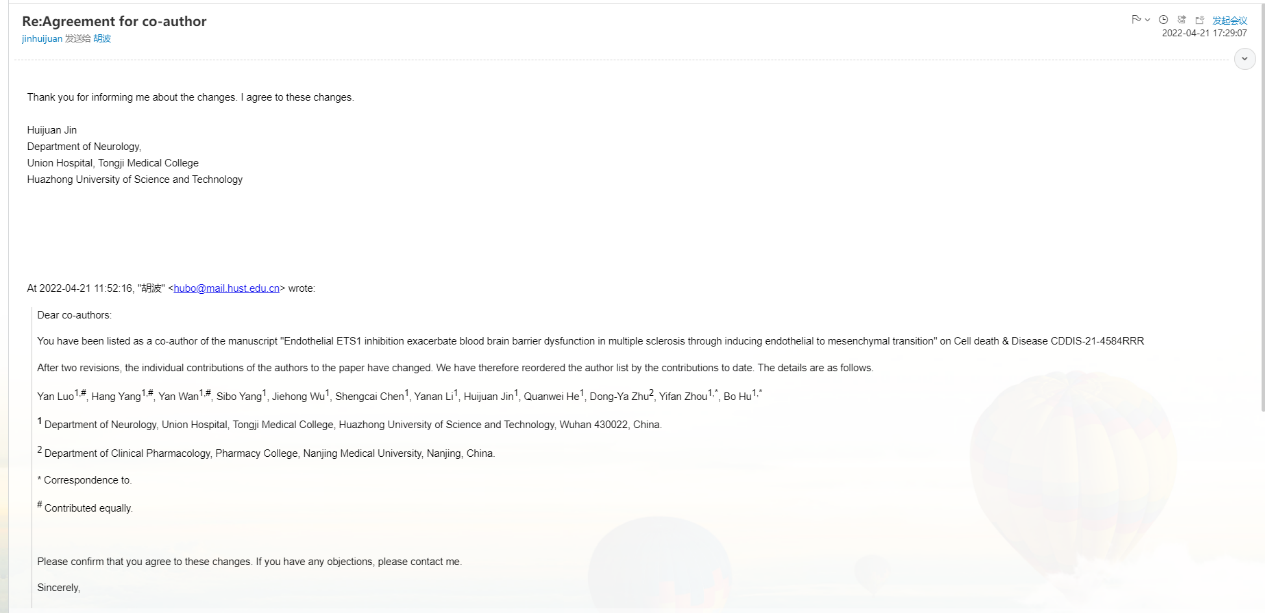 |
| Quan-Wei He | 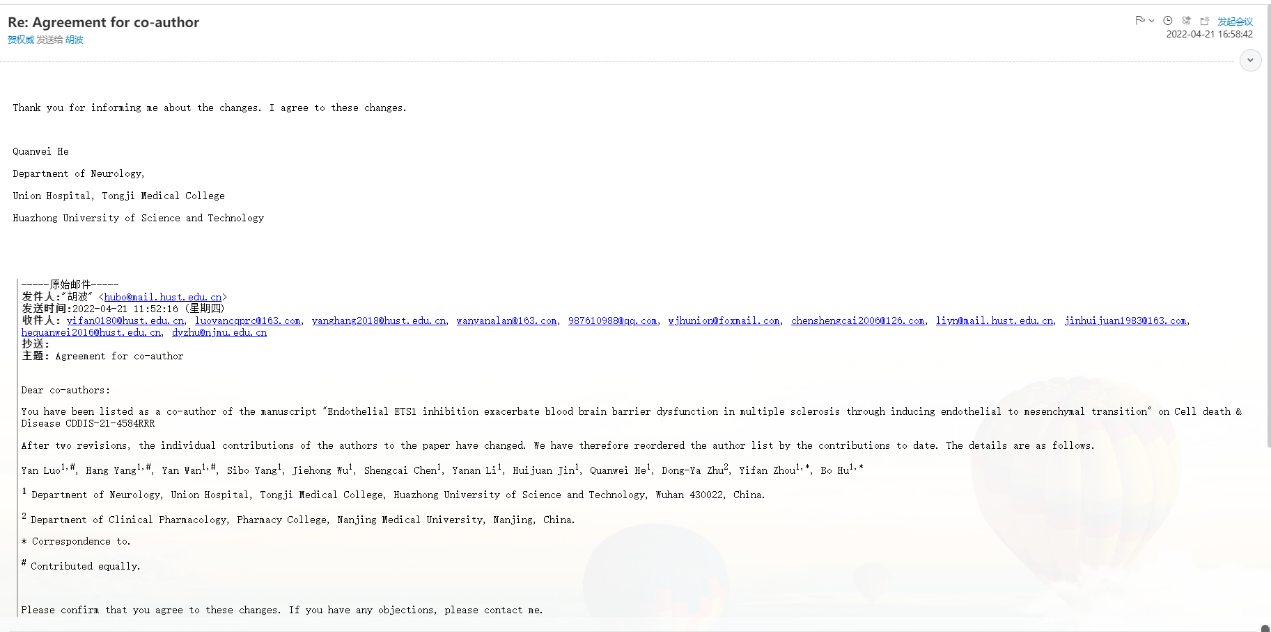 |
| Dongya Zhu | 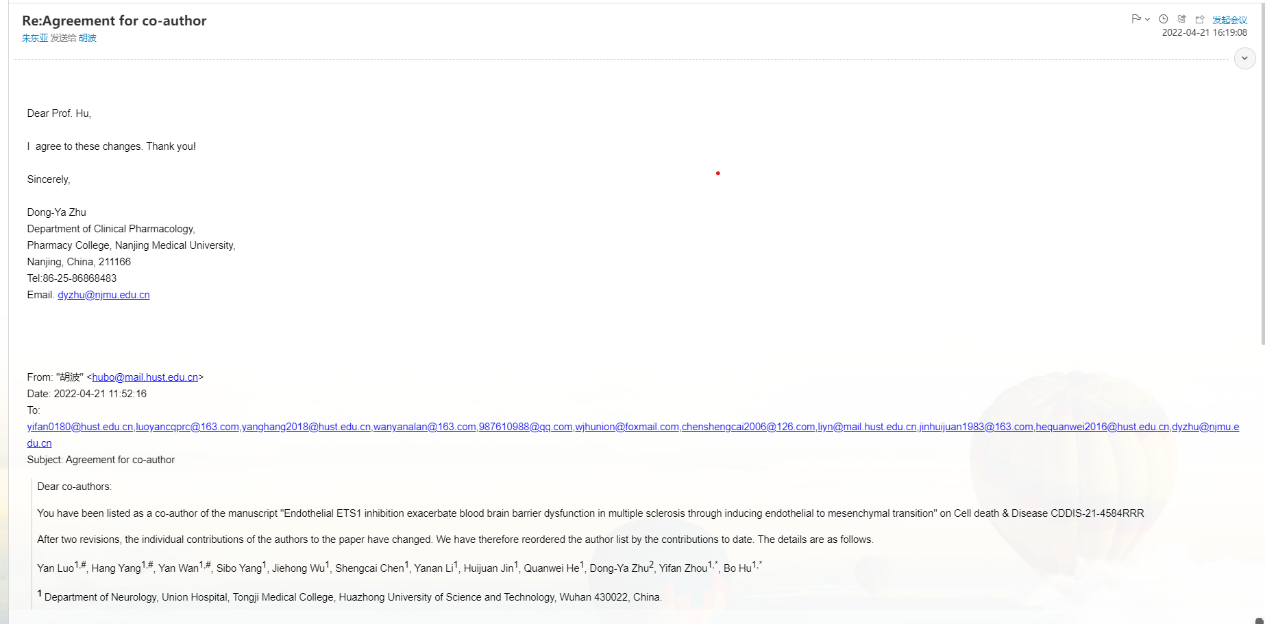 |
