# Supplementary material for: Endothelial ETS1 inhibition exacerbate blood–brain barrier dysfunction in multiple sclerosis through inducing endothelial-to-mesenchymal transition
Source: Cell Death Dis. 2022 May 14;13(5):462. doi: 10.1038/s41419-022-04888-5 (PMC9107459; doi:10.1038/s41419-022-04888-5)
Supplement: Supplementary file 5 — Supplementary Tables [file 41419_2022_4888_MOESM5_ESM.docx]

**Supplementary Tables**

**Supplementary Table 1. Primers for RT-qPCR Analysis**

| **Gene** | **Sequence 5′−3′** |  |
| --- | --- | --- |
| Vim | F: GGATCAGCTCACCAACGACA | |
|  | R: GGTCAAGACGTGCCAGAGAA | |
| Cdh2 | F: GCCGGAGAACAGTCTCCAAG | |
|  | R: CAAAGCTTCCGGGCGTAGA | |
| Cdh5 | F: CACGGACAAGATCAGCTCCT | |
|  | R: CACATAGTGGGGCAGCGATT | |
| Pecam1 | F: GTCCCCACCGAAAGCAGTAA | |
|  | R: GGTCAGGCTGGCCATTAACT | |
| Vwf | F: TGGGTTTTCTCTCCCTGGCT | |
|  | R: TTGCCCATACAAACAGGGGC | |
| Ocln | F: CCTGGTGTGAGCTGTGATGT | |
|  | R: GGAGAAGTCACCGCAGGAAA | |
| S100a4 | F: AGCACTTCCTCTCTCTTGGTC | |
|  | R: TTTTCCCCAGGAAGCTAGGC | |
| Tjp1 | F: TTCCGGGGAAGTTACGTGC | |
|  | R: AAGTGGGACAAAAGTCCGGG | |
| Acta2 | F: CCCAGACATCAGGGAGTAATGG | |
|  | R: TCTATCGGATACTTCAGCGGTCA | |
| Cldn5 | F: GTTAAGGCACGGGTAGCACT | |
|  | R: TACTTCTGTGACACCGGCAC | |
| Fn1 | F: ATGAGAAGCCTGGATCCCCT | |
|  | R: GAGAGCTTCCTGTCCTGTCT | |
| Gapdh | F: AGGTCGGTGTGAACGGATTTG | |
|  | R: TGTAGACCATGTAGTTGAGGTCA | |

**Supplementary Table 2. Antibodies for the Western Blot Analysis**

| **Name** | **Supplier** | **Cat No.** |
| --- | --- | --- |
| ETS1 | Cell Signaling Technology | 14069 |
| ETS1 | Proteintech | 12118-1-AP |
| CLDN5 | absin | abs130067 |
| FSP1/S100A4 | MilliporeSigma | 07-2274 |
| Vimentin | Proteintech | 10366-1-AP |
| N-cadherin | Proteintech | 22018-1-AP |
| Alpha-SMA | Servicebio | GB111364 |
| Occludin | Proteintech | 27260-1-AP |
| ZO-1 | Proteintech | 21773-1-AP |
| GAPDH | ABclonal | AC002 |
| Collagen Type I | Proteintech | 67288-1-Ig |
| Fibronectin | Proteintech | 15613-1-AP |
| VE-Cadherin | R&D Systems | AF1002 |
| CD31 | R&D Systems | AF3628 |
